# Exploring Chemical Spaces in the Billion Range: Is Docking a Computational Alternative to DNA-Encoded Libraries?

**DOI:** 10.1021/acs.jcim.4c00803

**Published:** 2024-09-21

**Authors:** Levente M. Mihalovits, Tibor V. Szalai, Dávid Bajusz, György M. Keserű

**Affiliations:** 1 Medicinal Chemistry Research Group and Drug Innovation Centre, HUN-REN Research Centre for Natural Sciences, Magyar tudósok krt. 2, 1117 Budapest, Hungary; 2 Department of Inorganic and Analytical Chemistry, Faculty of Chemical Technology and Biotechnology, Budapest University of Technology and Economics, Műegyetem rkp. 3., H-1111 Budapest, Hungary; 3 Department of Organic Chemistry and Technology, Faculty of Chemical Technology and Biotechnology Budapest University of Technology and Economics, Műegyetem rkp. 3., H-1111 Budapest, Hungary

## Abstract

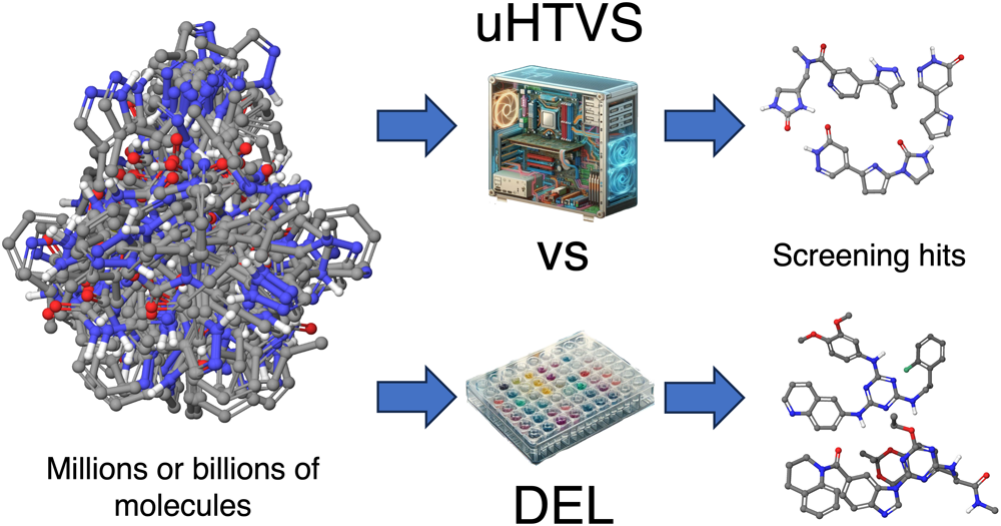

The concept of DNA-encoded libraries (DELs) enables the experimental screening of billions of compounds simultaneously, offering an unprecedented boost in the coverage of chemical space. In parallel, however, dramatically increased access to supercomputers and a number of ultrahigh throughput virtual screening (uHTVS) tools have made screening of billion-membered virtual libraries available. Here, we investigate whether current, brute-force, or AI-enabled uHTVS approaches might constitute a computational alternative to DEL screening. While it is tempting to look at uHTVS as a computational analogue of DEL screening, we found specific advantages and limitations of both methodologies that suggest them being complementary rather than competitive.

## Introduction

“Chemical space is vast”,^1^ to the extent that we need to define certain constraints to enumerate or even estimate the number of compounds it contains. In their pioneering work, Bohacek et al. estimated the number of compounds with ≤30 heavy atoms (a rather restrictive limit from a medicinal chemist’s point of view^2^) to exceed the 10^60^ order of magnitude.^3^ The past decade of innovation in medicinal chemistry and drug discovery was spearheaded by efforts to explore more and more of this vast universe, either experimentally or computationally.

In the experimental field, the concept of DNA-encoded libraries has appeared long ago,^4^ and its practical implementation was unlocked more recently by the necessary technological advances in e.g. robust polymerase chain reaction (PCR) amplification and DNA-sequencing techniques.^5^ DELs contain a vast chemical space of small molecules in a single vial, synthesized via combinatorial approaches and individually coupled to unique DNA sequences, acting as “molecular barcodes”.^6^ The encoding of each library member enables the simultaneous screening of the whole library: usually, the appropriately modified protein target is fixed on solid supports (e.g., streptavidin beads) and then incubated with the complete DEL library. Through a sequence of washing steps, most library members are removed from the solution, leaving only those with high affinity toward the protein target, which are then recovered by suitable elution procedures.^7^ The DNA-tags of these hit compounds are then decoded using PCR amplification and high-throughput DNA sequencing, after which the best hits are usually validated via off-DNA synthesis and/or orthogonal on-DNA assays.^8^

Besides its advantages and revolutionary role as a powerful hit discovery methodology, DEL screening comes with its unique challenges, mostly regarding the noise level during the selection and detection of binders, originating from several factors that might include the use of detergents, impaired folding of the target protein on the solid support, or nonspecific binding with the assay matrix.^9^ In general, the DNA tags of the hit compounds are enriched by PCR, followed by a fluorescent readout detection,^6^ and the number of reads for each tag is referred to as the “read count”. In theory, read counts can be correlated to binding affinity, with a higher read count accounting to a higher binding affinity; however, this does not necessarily translate into practice, as variable reaction yields and sequencing noise also influence read counts.^10^ To account for these technical effects, normalized read counts (Fn) can be used, which is calculated by taking the read count for a DNA tag and dividing it with the total number of read counts in the given sample.^11^ Just as stated before, denoising is important to process DEL data and detect binders. Available methods to denoise data include tagFinder,^12^ deldenoiser,^10^ and DEL-dock.^13^ The tool tagFinder is used for analyzing DEL data, accommodating technological errors, and enhancing compound identification and quantification.^12^ Deldenoiser is a method using sparse learning to address confounding factors in DEL sequencing data, significantly improving the robustness of hit identification, binder ranking, and structure–activity relationships.^10^ DEL-dock uses ligand-based descriptors and 3-D spatial information from protein–ligand complexes to denoise DEL count data.^13^

We have recently reviewed the current state of the art in the virtual screening of large compound libraries.^3^ Here, the main driving force of innovation in the past decade was, likewise, to access larger and larger chemical spaces. Significant efforts were dedicated to creating large databases of not yet synthesized but “synthetically accessible” (or “make-on-demand”) compounds by combining widely available starting materials via robust chemical reactions.^14,15^ The front runners of these efforts were compound vendors and aggregators like Enamine, Mcule, or eMolecules, providing access to billions of synthesizable compounds.^16−18^ In the meantime, more and more efficient “ultrahigh throughput virtual screening” (uHTVS) algorithms and workflows were published to enable the virtual screening of these expanded chemical spaces (10^8^+ compounds). These include GPU implementations of popular docking programs such as AutoDock (20–300× speedup vs CPU),^19^ as well as integrated workflows, such as Deep Docking,^20^ which delegates most of the docking computations to an iteratively refined deep learning model, or V-SYNTHES,^21^ a synthon-based concept for combinatorial library generation and docking. Similar speedups were reached for shape screening with the GPU-based version of the popular ROCS algorithm^22^ and for pharmacophore screening with the Pharmer method, which relies on efficient indexing techniques.^23,24^

Despite the high throughput, these developments come with certain trade-offs: for docking, the computational costs are still large, as the more efficient algorithms must filter through larger and larger data sets; therefore, the typical runtimes are still in the domain of days to weeks for a single virtual screening run on peak infrastructures (>27,000 GPUs for brute-force docking).^25^ An alternative to brute-force approaches is to introduce a logical layer into the workflow. Two main concepts were developed in previous years. The V-SYNTHES workflow integrates combinatorial library generation and docking: the screening deck is defined as the combination of a manageable number of molecular building blocks, which are first docked on their own, and then only the best initial hits are progressed into iterative growing and docking cycles.^21^ By contrast, several other workflows (including the aforementioned Deep Docking) leverage the computational demand of docking by using a small subset of the chemical space to train and iteratively refine a deep learning model to predict the docking scores, finally yielding a manageable number of virtual hits to be progressed into more sophisticated docking or even higher-demand calculations.^20,26,27^

Here, we investigate whether current uHTVS methodologies can democratize the access to ultralarge chemical spaces in an analogous way as virtual screening (VS) enabled economic hit discovery for small enterprises and academic groups two decades ago. Starting from our methodological comparison of experimental high-throughput screening (HTS) and VS in 2005^28^ and building our way upward through three case studies of DEL screening with different-sized libraries (from 10^5^ to 10^8^ compounds), we first identify some major methodological differences that challenge our comparison and then formulate conclusions and general guidelines for practitioners. Recognizing some bottlenecks of the software packages that are currently available for ultrahigh throughput virtual screening (e.g., time lost at the suboptimal structuring and parallelization of file conversion, ligand preparation, and other auxiliary tasks), we publish the scripts we developed in-house for conducting the screenings and analyses reported here: these are available open-source at https://github.com/keserulab/uHTVS_toolkit. Finally, we must stress that using DEL data sets as case studies in the present work serves merely a technical purpose in evaluating the two concepts against each other (i.e., which is the more effective way to explore chemical space). Namely, there are basically no other large data sets (apart from DEL screening data) on which uHTVS could be benchmarked/validated, as DEL screening provides an experimental result for all compounds (incl. inactives) of the tested chemical space. Prospectively, virtual screening of DEL libraries would have little added value due to the technical simplicity and speed of DEL screening itself. Nonetheless, uHTVS workflows can provide an additional layer for off-DNA compounds selection by docking specific parts of a DEL data set, acquiring information about which molecules are worth synthesizing off-DNA.

## Methods

### Data Sets

Three data sets were examined, labeled DEL-0,^29^ DEL-A, and DEL-B.^30^ The DEL-0 data set consists of saturated N-heterocycles. For the library, eight scaffolds were used: all four stereoisomers of 2,3-disubstituted azetidine and all four stereoisomers of 2,3-disubstituted pyrrolidine compounds. Appendages were selected based on physicochemical properties and conversion of the reaction used for synthesis, and overall, a total of 114 N-capping elements and 118 Suzuki-derived elements were chosen, resulting in the final library containing 107,616 distinct compounds (8 × 114 × 118). The quality of the DEL-0 data set was assessed by performing test screening experiments on horseradish peroxidase (HRP) and carbonic anhydrase IX (CAIX). HRP plays a major role as a reporter enzyme in diagnostics and histochemistry, and recently, it has been used in biocatalysis, bioremediation systems, and cancer treatment.^31^ CAIX is involved in pH regulation and potentially influences cell proliferation, cell adhesion, and tumorigenic processes.^32^ To address the nonuniform distribution of library barcodes and potential compound binding to the immobilization matrix, first a “barcode enrichment” score was developed by comparing a barcode’s abundance after screening to its abundance in a control without a target. However, this score was less reliable with low input representation, so the ratios between confidence limits (CI_95_) estimated from Poisson distributions based on observed sequence reads were calculated. The parameter “normalized fold change” (or normalized fold number, Fn) was defined as the ratio of a CI_95_ underestimate of the “after” distribution and a CI_95_ overestimate of the “before” distribution. Compounds were ranked by normalized fold change, and those with a normalized fold change value of at least 1 were treated as hits. Off-DNA synthesis was also done for six on-DNA positives from the CAIX screening and were subjected to a carbonic anhydrase inhibition assay.^29^

Production of the DEL-A and DEL-B data sets was started by acylating 192 Fmoc-amino acids to a double-stranded DNA headpiece, and after deprotection, a triazine was installed with cyanuric chloride. For the DEL-A data set, the remaining two chlorines on the triazine ring were substituted stepwise with 192–192 amines at each position, giving a total of 7,077,888 compounds (192 × 192 × 192) in the data set. Among the first 192 amines substituted, 3-amino-4-methyl-*N*-methoxybenzamide (AMMB) was included, which is a known pharmacophore fragment for p38 mitogen-activated protein kinase (MAPK). For the DEL-B data set, one of the chlorines on the triazine ring was substituted with 32 bifunctional acids, on which amidation was done with 384 amines, while the other chlorine on the triazine ring was substituted with 340 amines, in the end giving a total of 802,160,640 compounds (192 × 32 × 384 × 340) in the data set. Experiments were done against Aurora A kinase (AurA) for DEL-A and against MAPK for both DEL-A and DEL-B data sets. Aurora kinases are essential for the onset and progression of mitosis, with AurA promoting centrosome maturation and mitotic spindle assembly and playing tumor-promoting roles unrelated to mitosis, which includes tumor stemness, epithelial-to-mesenchymal transition, and invasion.^33^ MAP kinases have a role in coordinating cellular responses to nearly all stressful stimuli and are also involved in embryo development, immune responses, cell cycle, cell differentiation, cell metabolism, cell senescence, tumorigenesis, cell survival, and apoptosis.^34^ To select the hit compounds, PCR and DNA sequencing was used, and compounds with an insufficient number of copies (less than ten for DEL-A against MAPK, less than two for DEL-B against MAPK, and less than four for DEL-A against AurA) were filtered out. A control compound was also used as a control for the PCR artifacts during sequencing. For the DEL-A data set, off-DNA synthesis and a biochemical assay were done for three compounds (all containing AMMB) from the MAPK experiment and for seven representative analogues of the hit compounds from the AurA experiment. For the DEL-B data set, off-DNA synthesis and a biochemical assay were done for one on-DNA hit and four of its analogues from the MAPK experiment, all containing the benzimidazole-5-carboxylate substructure.^30^

### Enumeration and Preprocessing of the Libraries

#### DEL-0 Data Set

The first DEL data set was extracted from the work of Gerry and co-workers.^35^ SMILES strings were taken from the related supplementary material (compounds labeled as DEL) and were preprocessed with Schrödinger’s LigPrep at pH 7.4.^36^ Only one structure was written out per ligand. The data set consisted of 108,528 compounds by 8 scaffolds, 114 building blocks in position 1 (BB1), and 119 building blocks in position 2 (BB2). The 108,528 compounds are in fact 107,616 unique compounds, as BB2 70 and 77 are the same fragment in the original data set. However, experimental measurements show different values for these entries; hence, we handled the duplicated compounds separately, even though their docking scores were the same. Binding to the targeted proteins – carbonic anhydrase IX (CAIX) and horseradish peroxidase (HRP) – was evaluated by normalized fold numbers (Fn). On-DNA hits were classified as compounds possessing Fn equal to or larger than 1 measured for CAIX or HRP, separately. Off-DNA hits were collected from the publication, and the synthesized compounds were identified based on the constituting building blocks.

#### DEL-A Data Set

The second DEL data set was extracted from the publication of Clark et al.^30^ The DEL-A set consists of three building blocks, each with 192 different molecules, totaling 7,077,888 compounds. Here, in the absence of any compound identifier, enumeration was carried out using Marvin software.^37^ The building blocks were imported into MarvinSketch,^38^ the protecting groups were removed, link atoms were set, and the R-groups were constructed according to the building block positions. The batch mode of Markush Enumeration was used with the command line tool of Marvin’s cxcalc^39^ to create the SMILES strings for the compounds of the complete DEL-A library. Compound identifiers were created by the software as well. The enumerated compounds were preprocessed with LigPrep^40^ using similar settings as described for the DEL data set. Compounds of the DEL-A data set that possess experimental read count values were considered as hits. As the supporting material of the related publication contains only building block information on the hit compounds, hit identification was carried out by reconstructing the original compounds and then identifying them using the RDKit software package^41^ and the KNIME platform.^42^

#### DEL-B Data Set

The third and largest data set was also extracted from the work of Clark et al.^30^ Here, four building blocks constitute each compound in the DNA encoded library. The building blocks are grouped as Fmoc protected amines, bifunctional acids, and two distinct groups of amines, with members of 192, 32, 340, and 384 fragments, respectively. Here, the four building blocks constitute a DEL of 802,160,640 compounds. Enumeration was carried out as described in the DEL-A section using Marvin software. Due to the large number of compounds in the DEL-B library, the preprocessing of the molecules was carried out differently than the DEL-0 and DEL-A data sets. For further information, refer to the next section of the manuscript. Compounds of the DEL-B data set that possess experimental read count values were considered as hits. Hit identification was carried out the same way as described in the DEL-A section (see Table 1).

**Table 1:** Summary of the Collected and Analyzed DNA Encoded Libraries

Library name	Library size (compounds)	Target	Number of on-DNA hits	Number of off-DNA hits
DEL-0	108,528	CAIX	13020	6
		HRP	4595	-
DEL-A	7,077,888	MAPK	1855	0 (3)^a^
		AurA	207	0 (7)^a^
DEL-B	802,160,640	MAPK	18	1 (5)^a^

a The number in the parentheses shows the total number of compounds that have been synthesized off-DNA and evaluated by IC_50_ measurements, including close analogs of original on-DNA hits that were not included in the DEL library itself.

### Docking and Virtual Screening

#### Initial Assumptions

For each data set, the orthosteric binding site of the targeted proteins was used as the center of the docking grids. Allosteric binding regions were left out for the following reasons: (1) there are no known allosteric sites of CAIX, (2) for HRP, allostery was observed, but the allosteric site was not described unambiguously, (3) MAPK and AurA hits were primarily detected with an ATP-binding assay, and the reported crystallographic structures contained actual DEL members bound into the ATP (orthosteric) binding site of the kinases.^30^

#### DEL-0 Data Set

Our objective with this relatively small library was to explore the scope and limitations of evaluating a DEL data set by virtual screening. Therefore, for the DEL-0 data set, various screenings were carried out using different docking methods against both CAIX and HRP using the PDB structures 6NLV(43) and 7ATJ,^44^ respectively. The protein structures were preprocessed using the Schrödinger Protein preparation wizard.^45^ Grid generation was carried out for all Schrödinger-related docking jobs using Glide.^46,47^ A 4.5 Å spherical positional constraint was added for both grids at the solvent-exposed entrance of the binding sites. The ligands found in the PDB structures were set as the center of the grid box, while other settings were left at their default values. Glide docking was carried out using the SP (single precision) and HTVS (high-throughput virtual screening) modes, separately; additionally, a constrained SP docking was also performed (SPcon), where the linker group of the DNA tag was forced to occupy the previously defined outer face of the binding site. Docking with AutoDock (AD) was performed by using a specific workflow. Ligand preparation was carried out with the RDKit software,^41^ while protonation states were evaluated with the open-source program Dimorphite-DL.^48^ Grids for the docking with AutoDock were generated with AutoDockTools and AutoGrid.^49^ The crystallographic ligand was set as the center of the grid box. The docking was performed with the GPU implementation of AutoDock, AutoDockGPU,^19^ using an in-house script, that also creates the required pdbqt files of the separate ligands *in situ*. In the case of CAIX, the ligands were also docked using the AutoDock4_Zn_^50^ force field (AD_Zn_).

#### DEL-A Data Set

The DEL-A data set was virtually screened against AurA and MAPK using the PDB structures 3HA6 and 3HA8,^30^ respectively. The protein structures were preprocessed by the Protein Preparation Wizard.^45^ Both the Glide and AutoDock related grids were created with default settings, and the ligand of the X-ray structure was set as the center of the grid. The complete DEL-A library was docked into both proteins’ binding site using Glide^46,47^ with the HTVS precision. Additionally, Deep Docking^20^ was also used, coupled with both Glide HTVS and AutoDockGPU.^19^ Ligand preparation was carried out for the Deep Docking workflow as previously mentioned, using RDKit^41^ and Dimorphite-DL.^48^ Each Deep Docking workflow consisted of 11 cycles, each with 10,000 compounds to be docked in a single cycle (note that in the first iteration, 3 × 10,000 compounds are docked as training, test, and validation sets). At the end of the workflow, all of the prospective virtual hits based on the scoring of the trained machine learning (ML) model were written out. The complete AI-enabled docking was performed by using in-house python scripts. To complement the virtual screening results, the docking scores of the top 100,000 compounds based on Virtual Hit-Likeness (VHL) values were compared to 1,000, 10,000, and 100,000 randomly selected compounds. These amounts represent actual amounts of compounds that are feasible to dock in a routine case on moderate hardware.

#### DEL-B Data Set

Screening of the DEL-B data set was carried out for MAPK using the Deep Docking(ref (20))-Glide(refs (46 and 47)) HTVS combination using the previously described in-house scripts. Eleven Deep Docking cycles were performed, with each cycle using 1,000,000 compounds to train the ML model. At the end of the workflow, the prospective virtual hits were written out, and the best 100,000 compounds (based on the VHL-value) were docked into the previously defined binding site of MAPK using HTVS precision. To complement the virtual screening results, the docking of 100,000 randomly selected compounds was also performed.

#### Building Blocks

In the case of all three DEL data sets, we performed the docking of the building blocks separately. The building blocks were preprocessed with LigPrep once the protecting group was removed from the first building block position. The building blocks were then docked into previously defined binding sites (CAIX, HRP, AurA, MAPK) using Glide^46,47^ SP with default settings.

### Evaluation and Metrics

Compounds in the data set exported from the work of Gerry et al. with Fn larger than or equal to 1.0 were classified as on-DNA hits. To test the performance of the various virtual screening methods (SP, Spcon, HTVS, AD, AD_Zn_) on DEL screening, the receiver operating characteristic (ROC) curves were evaluated for each target-method pair, and also enrichment factors (EF) belonging to the best 0.5, 1, 2, and 5% of compounds ranked by docking score were calculated along with the area under the curve (AUC) values of the ROC curves. Where the ROC curve did not reach 100% for both axes, partial area under the curve (pAUC) values were calculated. Similar analysis was conducted based on the off-DNA hits: six compounds possessing nanomolar IC_50_ against CAIX. The relative positions of the hits based on their docking scores were also evaluated. In addition to the analysis of the complete molecules, building block distributions and building block compositions of both the experimental and virtual screening hits (best 100, 1,000 and 4,595 (HRP) or 13,020 (CAIX) compounds based on docking scores) were carried out.

Furthermore, the *P_bind_* score and compatible partners of the various building blocks in the separate positions were also calculated according to the work of Zhang.^51^ To calculate the *P_bind_* of a given building block, we define an S subset of the library, where we consider all of the compounds that contain that particular building block at a specific position. For example, we select building block no. 2 at the second building block position; then, the S subset contains all of the compounds that contain this specific building block at the second position. After the given building block is fixed, we calculate the *P_bind_* value as follows
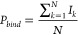
1where *N* is the size of the S subset, and *I*_*k*_ is 1 if the *k*^*th*^ compound in S is a hit and 0 otherwise. This calculation is carried out for every specific building block, separately. As the absolute *P_bind_* values are obviously dependent on the number of hits, we used the range scaled (normalized between 0 and 1) *P_bind_* values to compare the experimental and computational *P_bind_* values of the building blocks.

Compatible partners (CP) of a given building block were calculated as the number of building blocks that were found in combination with the given building block in a specific position among the hit compounds. Hence, the maximum number of compatible partners is the sum of the possible building blocks in the nonfixed positions. For example, we take the previous subset S, where all of the compounds contain building block no. 2 in the second position (BB2#2). Then, the number of compatible partners of that building block would be calculated as
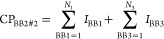
2where BB1 and BB3 denote the serial number of the building blocks in the specific positions, while *I*_BB1_ and *I*_BB3_ are 1 if the related building blocks can be found among the hit compounds with building block no. 2 in the second position and 0 otherwise. *N*_1_ and *N*_3_ are the total number of possible building blocks at the first and third positions.

### Deep Docking Workflow Evaluation

At the end of a Deep Docking workflow, it is possible to predict hits from the data set using the best trained model at the last iteration. The model predicts the virtual hit-likeness of all prospective virtual hits in the chemical library, which is termed as the p-value.^52^ The p-value or, as we labeled it, Virtual Hit-Likeness-value (VHL-value) is a number between 0 and 1, with a larger number meaning that the molecule is more virtual hit-like. We also investigated molecules with a VHL-value of at least 0.95, which serves as a stricter filter for the Deep Docking workflow. The prospective virtual hits were compared with the true hits from both data sets. Benchmarking statistics of the best model such as recall, precision, F_1_-score, and ROC-AUC were also evaluated after all iterations. Recall, precision, and F_1_-score values are defined as follows:
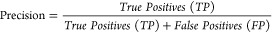
3
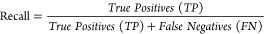
4
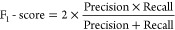
5

For more details regarding the benchmark statistics, please refer to Supplementary Note 1.

#### Accessibility Scoring

When docking DEL library members, a potential problem with the resulting binding poses is that the docking procedure does not account for the DNA tag (or the linker), meaning that the resulting binding pose might present the exit vector (i.e., the attachment point of the linker and DNA tag) in a buried position within the binding site, making the respective binding pose physically unavailable for the full (DNA-tagged) compound. To assess the validity of the poses found by various docking algorithms, the physical accessibility of the exit vectors was evaluated using the following equation:
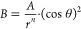
6Eq 6 defines a repulsive potential between atoms of the ligand and the protein target, with the distance *r* between the end point of the exit vector (terminal ligand atom) and the protein atom and the angle θ of the exit vector against the line connecting the terminal ligand atom with the protein atom. *A* and *n* are scaling factors (set to 10^9^ and 6, respectively) that control the recline of the repulsive potential *B*, which is referred to as the accessibility score from here on. A higher value of *B* means that the examined atom is less accessible from the solvent phase, while with *B* = 0.0, the examined atom is fully accessible, the DNA tag can occupy the space at the opening of the binding pocket, and thus, the pose can be considered valid for the given DEL compound. Exit vectors for the specific DEL data sets are summarized in Figure S1. In the case of multiple exit vectors, the one with the lower accessibility score was retained for evaluation. The ratio of feasible molecules was calculated using a KNIME workflow in which molecules possessing *B* = 0.0 were considered as feasible, while molecules with *B* > 0 were considered to exhibit a DEL-incompatible docking pose.

## Results and Discussion

Virtual screening was originally developed as a low-cost computational alternative to experimental high-throughput screening. With the more flexible screening decks and highly customizable workflow, virtual screens were reported with hit rates that vastly exceed those of HTS, reaching as high as 73% in exceptional cases.^53^ Our historic direct comparison of the two methodologies against glycogen synthase kinase-3β (GSK-3β) revealed a 12.9% hit rate for VS, in contrast to 0.55% for HTS^28^ from the same compound deck.

With the expansion of the available chemical spaces beyond what is manageable even by computers, we are faced with new challenges in comparing the respective successors of these methodologies, DEL screening, and uHTVS. Specifically, the primary hit detection platform of DEL screening provides noisier data and in general less reliable hit identification than classical HTS platforms. At the same time, AI-enabled uHTVS workflows submit only a small portion of the screening deck into the actual docking stage, while replacing the docking of most of the data set with the less expensive deep learning model. Here, we will address these challenges step by step: we first explore the smaller DEL-0 library screened against horseradish peroxidase (HRP) and carbonic anhydrase IX (CAIX) by Gerry et al.,^35^ benchmarking the performance of several docking algorithms in a brute-force setting. Then, we compare brute-force vs AI-enabled virtual screening on the larger DEL-A library. Finally, we benchmark the Deep Docking uHTVS workflow against the DEL-B library of 800+ M compounds.

### DEL-0 Data Set

We started our work focusing on the relatively small DEL-0 library extracted from the work of Gerry et al.^35^ with available experimental results for all the library members. Here, the number of compounds to be screened enabled the use of “brute-force” virtual screening, namely, the docking of every member of the given library. To achieve a comprehensive picture of how open-source and proprietary docking software performs, we selected AutoDockGPU (AD) and Glide as benchmarking tools. Glide jobs were performed using three different scenarios, namely using single precision (SP), HTVS precision, and single precision coupled with a positional constraint (Spcon) applied for the linker group of the DNA tag. First, we analyzed how the on-DNA hits can be recovered based on the docking scores. The ROC curves (Figure S2) show similar screening performance for all the applied methods. AutoDock seems to perform slightly worse than Glide; however, the open-source nature of AutoDock promotes it as a great alternative for proprietary docking software. Based on the enrichment factor values and the AUCs (Table 2) of the ROC curves, only a slightly better hit ratio can be obtained by screening the DNA encoded libraries virtually than selecting the compounds randomly. The lower enrichment factor can be attributed to the noisy nature of the experimental results and also to the subjectively selected Fn threshold, which was extracted from the original publication of Gerry et al. Glide docking jobs (SP, Spcon, and HTVS) seem to perform better than AutoDock; however, AutoDock was able to dock all of the library members into both binding sites. Interestingly, Glide HTVS was not able to accommodate around 40% of the compounds into the targeted site of HRP, owing to the small size of the protein’s binding pocket. However, the pAUC values of HTVS against the two targets show highly similar performance for both cases (Table 2). As CAIX is a zinc metalloprotein, we have also applied the AutoDock4_Zn_ force field to dock the DEL-0 library using AutoDockGPU.^50^ Interestingly, the docking performance has not been improved in terms of enrichment factors and pAUC compared to the default force field of AutoDock.

**Table 2:** Summary of the Docking of the DEL-0 Data Set

Method	Target	EF_0.5%_	EF_1%_	EF_2%_	EF_5%_	pAUC^a^	Time Requirement^b^ (s)
SP	CAIX	1.274	1.305	1.256	1.261	0.537	1,339,376^c^
Spcon	CAIX	1.320	1.297	1.244	1.198	0.540	1,359,360^c^
HTVS	CAIX	1.474	1.405	1.359	1.292	0.533	80,380^c^
AD	CAIX	0.921	0.967	0.933	0.971	0.506	151,104
AD_Zn_	CAIX	0.906	0.906	0.902	1.024	0.497	152,752
SP	HRP	1.000	1.174	1.229	1.236	0.552	963,880^c^
Spcon	HRP	0.913	1.131	1.142	1.201	0.542	2,401,896^c^
HTVS	HRP	0.957	0.913	0.979	1.206	0.546	42,600^c^
AD	HRP	0.609	0.848	1.273	1.175	0.477	175,472

a Partial area under curve.

b Projected onto a single thread of CPU (SP, Spcon, HTVS) or GPU (AD).

c The total time requirement of Glide related jobs is increased by the time requirement of the library preparation with LigPrep, which is an additional 39,616 s projected onto a single thread of CPU. By contrast, ligand preparation is included in the time requirements reported for AD.

Apart from the predictive power, another important aspect of the docking methods is computational requirements. Glide jobs operate on CPUs, while AutoDockGPU uses GPUs. Hence, it is expected that AutoDockGPU outperforms the Glide jobs in terms of computational time per processing unit. The time requirement of the respective jobs projected onto a single CPU or a GPU are shown in Table 2. As anticipated, AutoDockGPU performs faster than Glide SP and Spcon jobs; however, it does not outperform Glide HTVS on a single CPU even when we consider the additional time requirement of ligand preparation. Moreover, a Glide HTVS job running on 8 CPUs would overwhelmingly outperform an AutoDockGPU job running on a single GPU; moreover, multiple CPUs are more feasible to access than multiple GPUs. As we used 8 CPUs to dock the compounds for the Glide SP and Spcon, 2 CPUs for the Glide HTVS, and 4 GPUs for the AutoDockGPU jobs, all of the virtual screenings finished in less than 2 days, underscoring that the brute-force virtual screening of a 100,000 member DNA encoded library is feasible even with relatively modest computational infrastructure.

**Figure 1:**
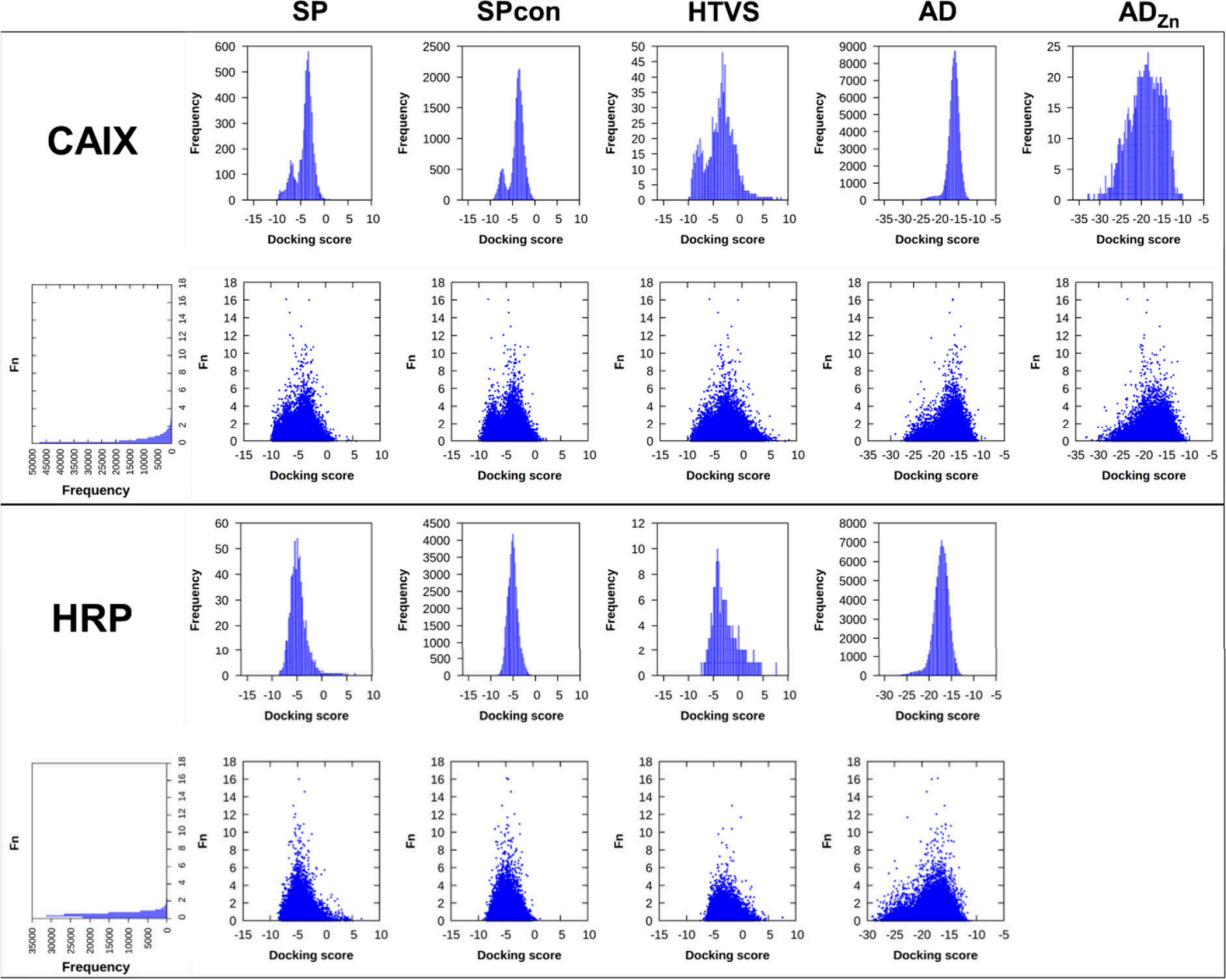
Comparison of normalized fold numbers and docking scores along with the distribution plots of the depicted parameters for the DEL-0 data set.

Next, we compared the docking score and Fn value distributions (Figure 1). It is clear that the correlation between the two measures is missing; the expected trend of decreasing docking scores coupled with an increasing Fn is absent. Interestingly, the distribution of the docking scores shows single (HRP) and multiple (CAIX) Gauss-like distributions. The latter is caused by the docking algorithm favoring specific building blocks and giving compounds with these building blocks better scores (Figure S3). The distributions of Fn values show a maximum at lower Fn values with a steep decrease thereafter. This is similar to what was observed in our earlier work,^28^ where virtual screening was compared to HTS.

To obtain a noise-free comparison between experimental and computational results, we evaluated the ROC curves based on the off-DNA hits (Table S1). We classified 6 compounds with IC_50_ values as hits, as **10a** and **10b** of the original publication turned out to be inactive against CAIX.

**Figure 2:**
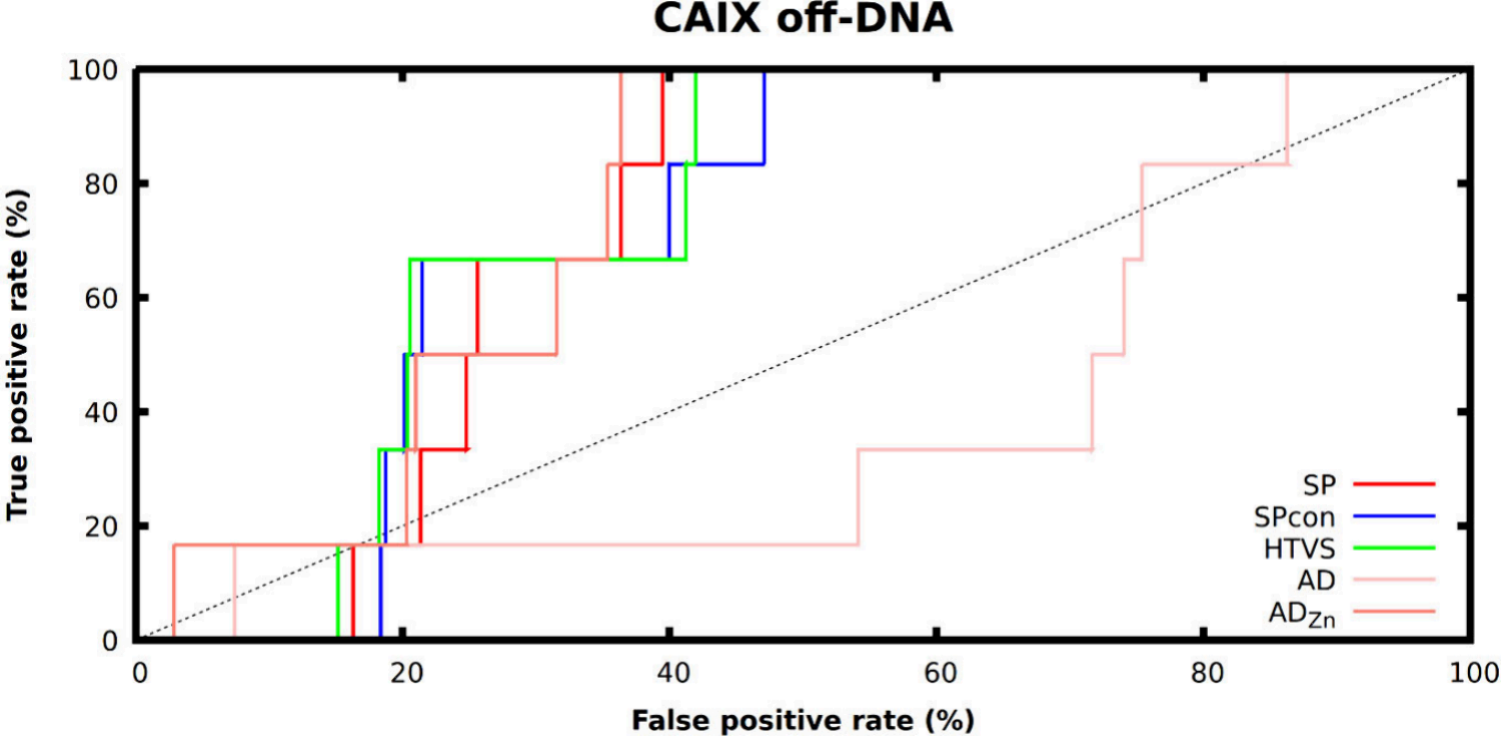
ROC curves of the virtual screening methods using the DEL-0 data set with off-DNA hit classification (AUC values: 0.726 SP, 0.724 Spcon, 0.725 HTVS, 0.385 AD, 0.754 AD_Zn_).

Here, AutoDockGPU with the standard AutoDock force field performed very poorly, while the modified AutoDock4_Zn_ resulted in similar performance as the Glide-related methods showing sensible AUC values (Figure 2). However, it is worth mentioning that the first hit has been reached after the best 15% of the ranked compounds in the Glide jobs, while AutoDock found the first hit compound among the best 8% of the ranked molecules both with the standard and AutoDock4_Zn_ force field. (In contrast, the second hit was only recovered in the second half of the data set by the default AutoDock force field.)

### DEL-A Data Set – Brute-Force Docking

**Figure 3:**
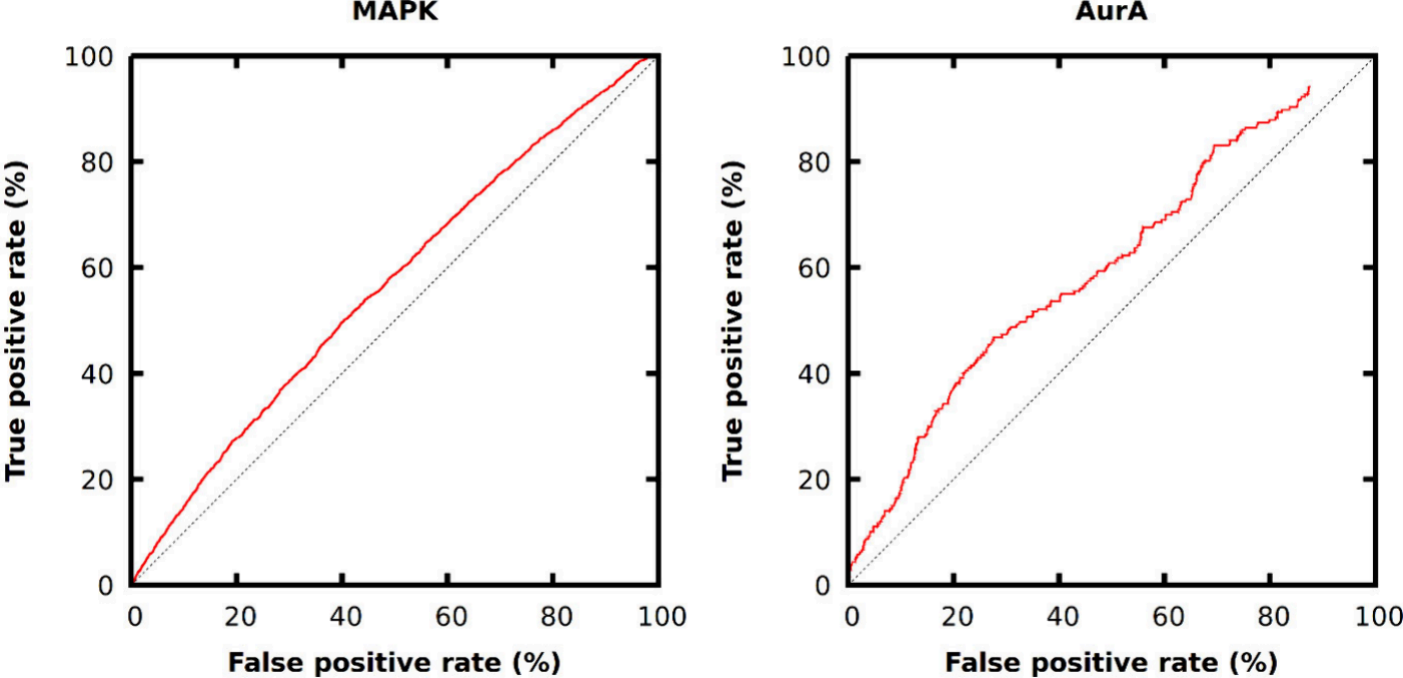
ROC curves of HTVS docking of the DEL-A data set against MAPK and AurA. Compounds are ranked by docking scores.

We have conducted a brute-force docking of the DEL-A data set, containing 7,077,888 compounds altogether against MAPK and AurA. The experimental hits were extracted from the results of Clark,^30^ and compounds with read count information were classified as hits. Due to the high number of compounds to be docked, only Glide HTVS docking was performed for both proteins. The ROC curves (Figure 3) show similar results to the DEL-0 data set of Gerry et al., with similar pAUC values. Here, Glide HTVS was able to accommodate more than 85% of the overall library for both proteins, probably due to the fact that the protein structures used for the DEL-A data set are actual DEL compound-protein complexes. The time requirement of the docking per single CPU ranges between 300 and 450 days; however, we performed the calculations on 48 CPUs in parallel, which reduced the time requirement to 6–9 days. Additionally, ligand preparation with LigPrep took around 5 days on 8 CPUs, meaning 40 days per single CPU. These results suggest that the brute force virtual screening of a 10 million compound DNA-encoded library with average computational infrastructure is still feasible; however, the docking results require further evaluation as they show modest performance in terms of enrichment factors. In the case of AurA, the first 1,000 ranked compounds did not contain any experimental hits (see Table 3).

**Table 3:** Summary of the Docking of the DEL-A Data Set

Method	Target	EF_0.5%_	EF_1%_	EF_2%_	EF_5%_	pAUC^a^	Successfully docked compounds	Percent of docked compounds (%)	Time Requirement^b^ (s)
HTVS	MAPK	1.402	1.941	1.725	1.596	0.560	6,934,278	98.0	36,056,546
HTVS	AurA	6.763	4.348	2.899	2.222	0.588	6,206,392	87.7	26,104,771

a Partial area under curve.

b Projected onto a single thread of CPU.

### DEL-A Data Set – Ultrahigh Throughput Virtual Screening (uHTVS) with an AI-Enabled Workflow

**Figure 4:**
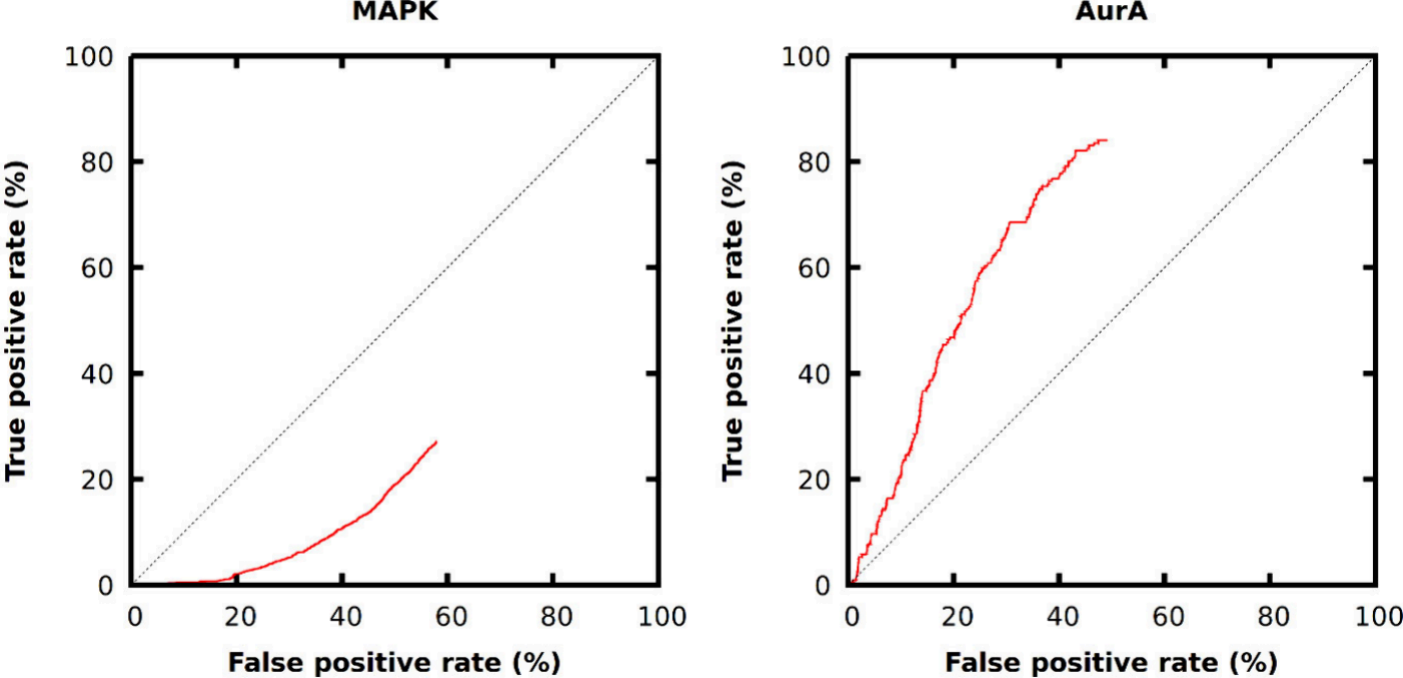
ROC curves of Deep Docking-Glide HTVS workflow prospective virtual hits of the DEL-A data set against MAPK (pAUC: 0.285) and AurA (pAUC: 0.606). Compounds are ranked by VHL-values.

The size of the DEL-A data set raised an opportunity to test the performance of an AI-assisted virtual screening workflow and compare it to the brute force all-ligand docking. We trained the AI-based Deep Docking model with the docking results of both Glide HTVS and AutoDockGPU against MAPK and AurA, using 1.8% of the complete library. The prospective virtual hits, ordered by their scores (VHL-value) given by the trained model, were compared to the experimental hits of the DEL-A data set (Table 4). The workflow with Glide HTVS performed differently for the two protein targets. In the case of MAPK, only 27.1% of the experimental hits were recovered, whereas this number is 84.1% for AurA. Here, the combination of AutoDockGPU with Deep Docking performed poorly: only four hits were recovered for MAPK, and none were recovered for AurA. These huge differences between the workflows can be attributed to the poor docking results of AutoDockGPU. A vast number of compounds cannot be docked using this technique; hence, the model is biased toward molecules that can be accommodated into the binding pocket by the AutoDockGPU code – compounds with larger or harder-to-fit building blocks are dropped out – and the number of prospective virtual hits at the end of the workflow is reduced to a great extent compared to the Glide-based workflows. This also correlates with the fact, that the Deep Docking results rely entirely on the suitability of the docking engine.^52^ The large number of prospective virtual hits using Glide HTVS is the other end of the scale, and around half of the complete library was labeled as a prospective hit for both targets, which is a questionable screening outcome. However, following a typical screening workflow, only a predefined number of prospective virtual hits should be written out. If we select the best 50,000, 100,000, and 1,000,000 prospective virtual hits based on the Deep Docking VHL-value, the number of experimental hits recovered are 0 (EF_50000_: 0.000), 0 (EF_100000_: 0.000), and 15 (EF_1000000_: 0.057), respectively, for MAPK and 1 (EF_50000_: 0.745), 2 (EF_100000_: 0.745), and 70 (EF_1000000_: 2.608), respectively, for AurA. This highlights that if both the brute-force and AI-assisted dockings are available for DEL docking, the brute force alternative is more effective. The ROC curves (Figure 4) show highly different performances against the two targets and do not approach the top right corner of the graph as observed for the brute-force methods. Here, only compounds labeled as prospective virtual hits are considered. The benchmarking statistics of the Deep Docking models (refer to Supplementary Note 1, incl. Figure S18) show significantly different precision values between workflows using Glide and workflows using AutoDockGPU. Low precision values in the case of Glide runs explain the large number of prospected virtual hits and false positives. In the case of AutoDockGPU runs, the precision values show local maxima at the sixth and eighth iterations. Notably, this suggests a better ML model after iteration eight than after iteration 11. Predicting virtual hits with the best ML model after iteration eight for both MAPK and AurA resulted in better experimental hit recovery in the case of MAPK, with 21 experimental hits recovered out of 94,714 prospected virtual hits, while it did not confer an improvement in the case of AurA (0 experimental hits within the 62,827 prospected virtual hits). With Glide against MAPK, the ROC-AUC value of the best ML model stayed the lowest compared to the other Deep Docking runs, even after iteration 11, and did not show an increasing tendency throughout the iterations, as was observed in the other runs. This suggests that the iterations were not able to improve the ML model, and it could also explain the poor performance observed in Figure 4. Regarding the time requirement of the workflows, Glide HTVS jobs were completed in 2–3 days on 48 CPUs, while the AutoDockGPU jobs were run for 3–4 days on 4 GPUs on average. These numbers are half of the time requirement of the brute-force methods; however, the difference between the performance of the two workflows still promotes brute-force docking instead of using Deep Docking.

**Table 4:** Summary of the Deep Docking Workflow Results on the Del-A Data Set against MAPK and AurA Kinase Applying Glide HTVS and AutoDockGPU

Docking method	Target	Prospective virtual hits	Recovered experimental hits (all hits)	Percent of recovered experimental hits (%)
Glide HTVS	MAPK	4,097,704	503	27.1
Glide HTVS	AurA	3,482,676	174	84.1
AutoDockGPU	MAPK	41,937	4	0.22
AutoDockGPU^a^	MAPK	94,714	21	1.13
AutoDockGPU	AurA	28,985	0	0.00
AutoDockGPU^a^	AurA	62,827	0	0.00

a Best model, after iteration eight.

The results become worse if we only take those Deep Docking virtual hits into account, that have VHL-values of at least 0.95, which reduces their number significantly (Table 5). This way, only 2-2 hits are recovered for AurA with Glide HTVS and for MAPK with AutoDockGPU as the docking engine, and none are recovered for the rest (AurA with AutoDockGPU and MAPK with Glide HTVS). These results suggest that filtering based on the VHL-value is only feasible if the number of prospective virtual hits is still too high for brute-force methods. For the distribution of VHL-values with the prospective virtual hits from the data set known as the activation function in neural networks,^54^ refer to Figure S4.

**Table 5:** Summary of the Deep Docking Workflow Results with Prospective Virtual Hits with a VHL-Value ≥ 0.95 on the DEL-A Data Set against MAPK and AurA Kinase Applying Glide HTVS and AutoDockGPU

Docking method	Target	Prospective virtual hits with VHL-value ≥ 0.95	Recovered experimental hits	Percent of recovered experimental hits (%)
Glide HTVS	MAPK	67,213	0	0.00
Glide HTVS	AuroraA	101,509	2	0.97
AutoDockGPU	MAPK	13,085	2	0.11
AutoDockGPU	AuroraA	13,784	0	0.00

**Figure 5:**
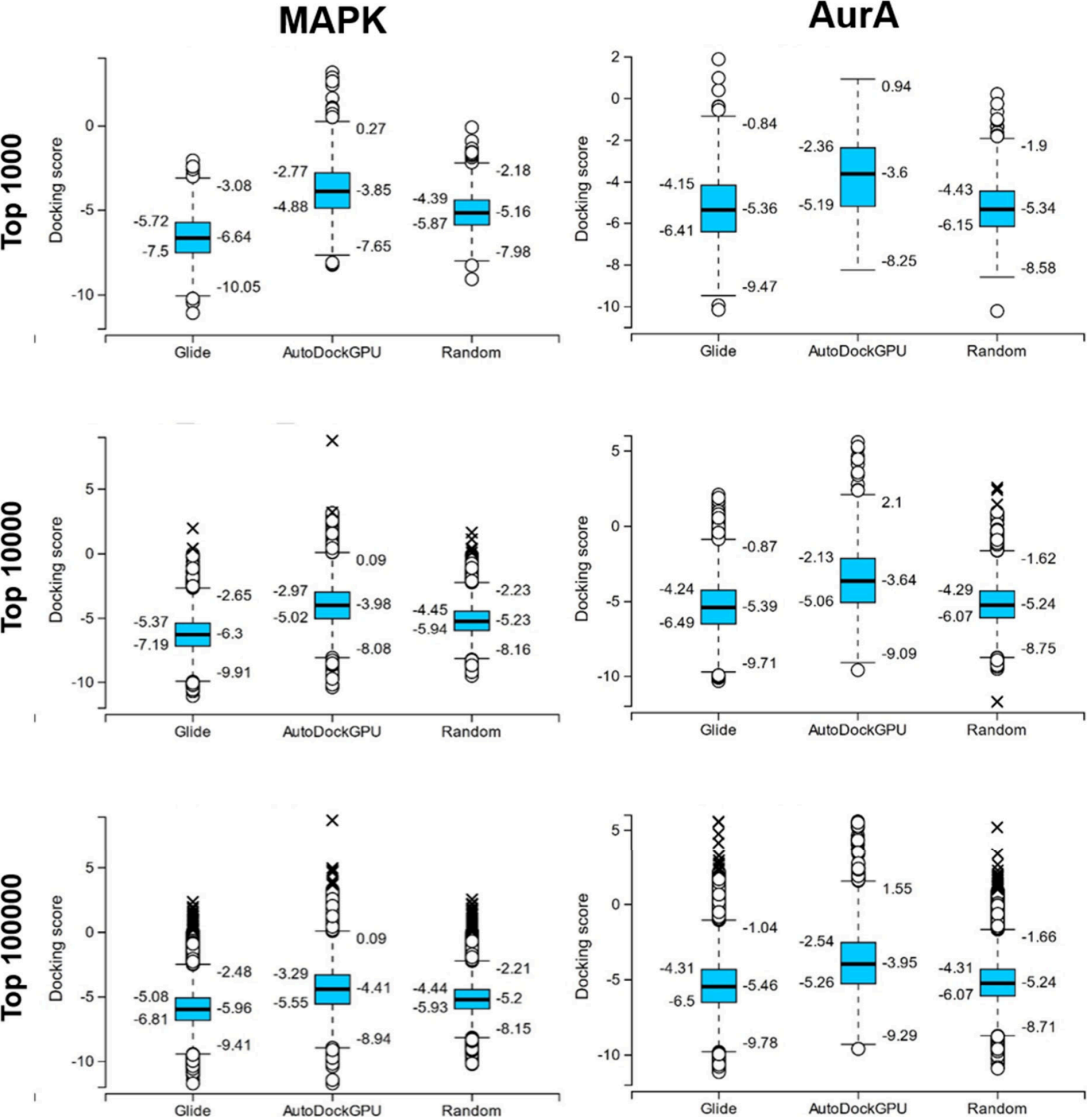
Box and whisker plots of the docking score distributions for the top 1,000 (plots on the top), 10,000 (plots in the middle), and 100,000 (or all prospective virtual hits, where that value was lower than 100,000) (plots at the bottom) compounds based on their VHL-value for Deep Docking workflows done using Glide and AutoDockGPU and the docking score distributions for 1,000, 10,000, and 100,000 compounds selected by random sampling from the DEL-A data set for AurA (left) and MAPK (right).

To compare the performance of the Deep Docking workflow against random sampling, 1,000, 10,000, and 100,000 compounds were randomly selected from the docked data set, and their docking scores were compared to the same number of top compounds from Deep Docking (or all the prospective virtual hits, if that value was lower than 100,000), based on VHL-values. For the amount of prospective virtual hits for each method-target pair, refer to Table 4. The resulting box and whisker plots are listed in Figure 5. It is important to note that for the present comparison, all compounds were evaluated by Glide HTVS (even the top ones resulted from the Deep Docking workflow done using AutoDockGPU), due to the generally better performance of Glide. Comparing the results with the docking score distribution acquired from random sampling, the figure shows that against AurA, the Deep Docking workflow with Glide produced almost the same docking score distribution but with a more negative lower extreme value, while using AutoDockGPU produced a worse docking score distribution (distribution is shifted toward more positive values). Against MAPK, the Deep Docking workflow with Glide produced a better docking score distribution (distribution is shifted toward more negative values, and the lower extreme is at a more negative value), while AutoDockGPU produced a worse docking score distribution than what was acquired from random sampling. These results partly contradict the results extracted from the ROC curves in Figure 4, as the methods with the more negative docking score distribution would have been the ones expected to produce better ROC curves, and here, it shows that Glide performed better against MAPK than AurA in terms of the docking score distribution compared to random sampling. However, it is also important to note here, that the ROC curves were plotted with the millions of prospective virtual hits, while the box and whisker plots were plotted with a maximum of 100,000 compounds. As expected, increasing the sample size did not change the distribution of the docking scores significantly for random sampling. For the Deep Docking workflow with Glide against MAPK, increasing the sample size shifted the distribution toward more positive values, and for the rest, it shifted the distribution toward more negative values. This shift based on the sample size suggests that the results with Glide correlate with the ROC curves in Figure 4, as getting closer to the full amount of prospective virtual hits should result in Glide against AurA to perform better and against MAPK to perform worse than random sampling.

### DEL-B Data Set – Ultrahigh Throughput Virtual Screening (uHTVS) with an AI-Enabled Workflow

**Figure 6:**
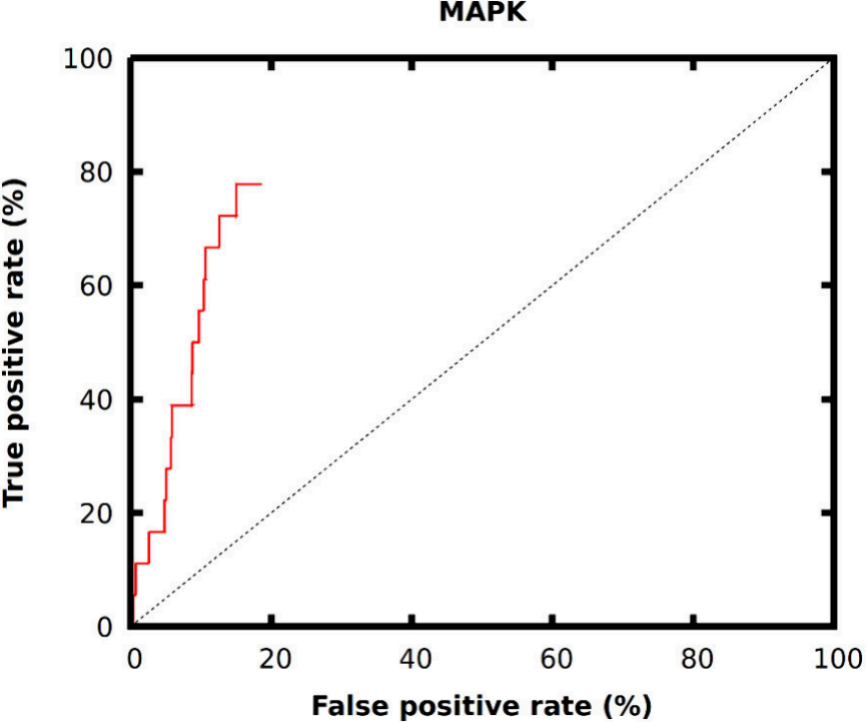
ROC curve of Deep Docking GlideHTVS workflow prospective virtual hits of the DEL-B data set against MAPK (pAUC: 0.612).

In the case of the DEL-B data set, the vast size of the DNA-encoded library enabled us to perform only the AI-assisted workflow coupled with Glide HTVS, which took slightly more than one month (around 38 days) using 48 CPUs. The docking results were compared to the experimental results, namely, to the compounds with experimental read counts classified as hits. The workflow consisted of docking 1.6% of the compound library to train the ML model; however, this meant the actual docking of 13 million compounds. Here, even the number of prospective virtual hits exceeds 150 million. A sensible threshold would filter out unlikely prospective virtual hits; therefore, we first examined the hits of the Deep Docking workflow that have a VHL-value ≥ 0.95 (1.8 million compounds). Unfortunately, none of the 18 experimental hit compounds were among the filtered prospective virtual hits, which is not surprising owing to the multiple orders of magnitude difference between the library size and the number of hits. However, upon examination of all the (unfiltered) prospective virtual hits, 14 out of the 18 experimental hits (77.8%) were among them, suggesting that it is not always a beneficial idea to filter out the prospective virtual hits based on the VHL-values. The ROC curve shows a promising performance against the MAPK target (Figure 6). The curve also shows a steep initial increase, with enrichment factor (EF) values of 11.1 at 1% and 5.56 at 2%. As the brute-force docking of this vast number of molecules would not be feasible, the molecules in the ROC curve are ranked based on their VHL-values.

The one experimental on-DNA hit that was synthesized off-DNA (compound **13** in the original publication of Clark et al.^30^) was also within the 14 hits identified by Deep Docking, having the 11th highest VHL-value out of the experimental hits.

**Figure 7:**
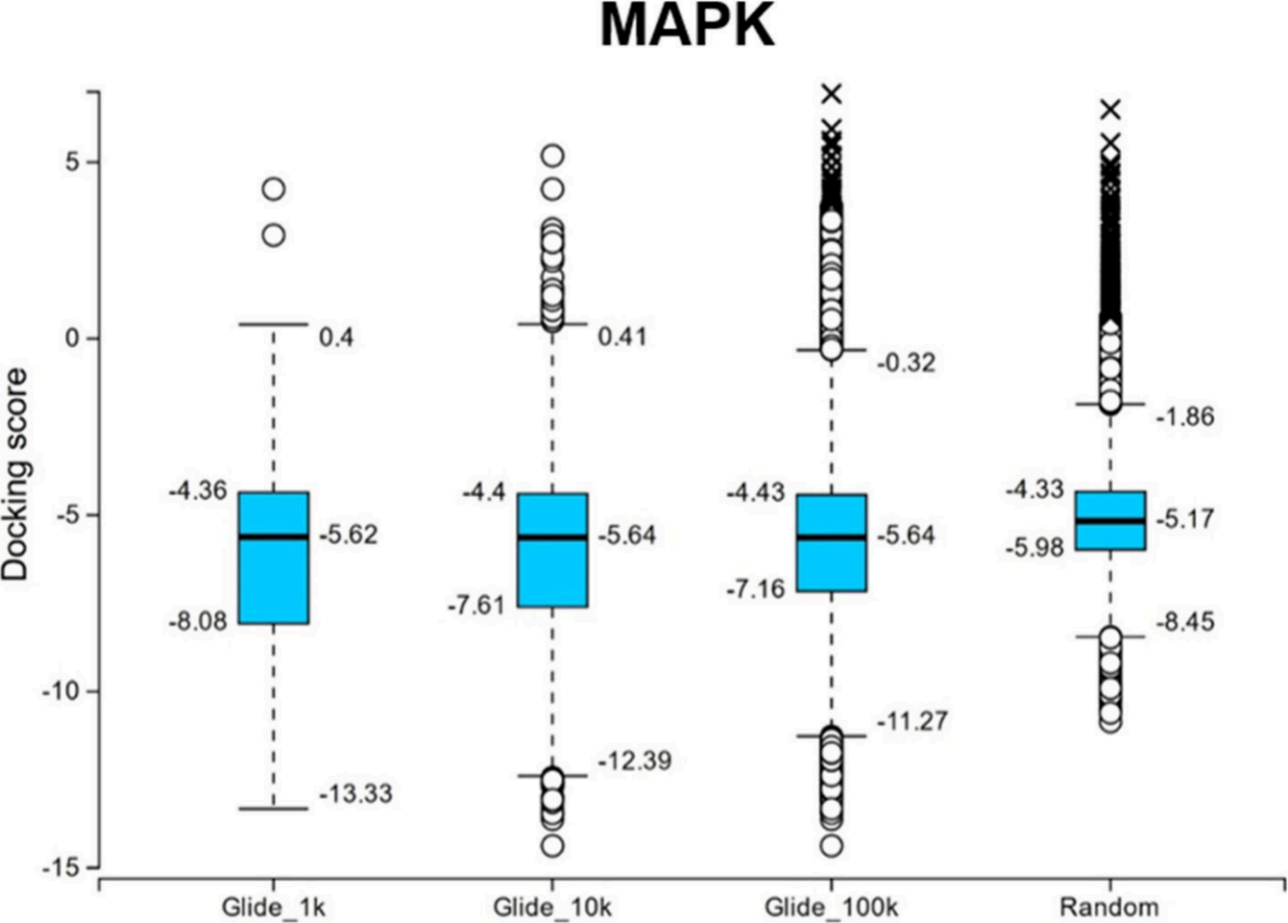
Box and whisker plots of the docking score distributions for the top 1,000 (Glide_1k), 10,000 (Glide_10k), and 100,000 (Glide_100k) compounds based on their VHL-value for Deep Docking workflows done using Glide and the docking score distributions for 100,000 compounds selected by random sampling from the DEL-B data set. All dockings were performed against MAPK.

Performance of the Deep Docking workflow against random sampling was also examined for the DEL-B data set. Here, 100,000 compounds were randomly selected from the data set and were docked with Glide HTVS. The top 100,000 prospective virtual hits based on VHL-values were also docked with Glide HTVS. The results are summarized in Figure 7. The docking score distributions show that the Deep Docking workflow with Glide achieved a docking score distribution more negative than that of random sampling. The results also show significantly more negative lower extremes compared to that from random sampling, which is advantageous, as the manual observation of the binding poses starts with the compounds having the most negative docking score values.

### Building Block Analysis – DEL-0 Data Set

We conducted a building block-based analysis and hit evaluation of the experimental and computational results as well. Instead of using a simple distribution of building blocks, we calculated the *P_bind_* values building block-wise according to the work of Zhang et al.^51^ We have chosen to use the normalized *P_bind_* values to be able to compare them for different sized subsets of molecules (see the “Evaluation and Metrics” subsection). In each case, a higher *P_bind_* value means that a larger proportion of experimental or virtual hits contains the considered building block in the specific position, i.e., that building block in that specific position confers an important contribution to on-target affinity. We have plotted normalized *P_bind_* values for different sized subsets of experimental and virtual hits (top 100, top 1000, and so on), based on the Fn values and docking scores (Figure S5). For CAIX, the BB1 position seems to tolerate several building blocks among the top 100 experimental hits but shows a clearer preference for 1–3 building blocks as we move to larger compound sets: in the meantime, the docking results show an opposite trend with 1–2 preferred building blocks in the top 100 sets of virtual hits vs several when we move toward bigger sets. The BB2 position shows an opposite trend with one clearly preferred building block among the top 100 and 1,000 experimental hits vs several in the top 13,020; meanwhile, the preferred building blocks are more diverse among the virtual hits all along. There is similarly little correlation between the trends among the experimental vs virtual hit sets for HRP, as well. Overall, we cannot argue that any of the docking programs would accurately retrieve the preferred building blocks among the experimental hits.

We also evaluated the number of compatible partners of the specific building blocks based on the best 100, 1,000, and 13,020 compounds against CAIX and the best 100, 1,000, and 4,595 compounds for HRP (Figure S6). Similar to the *P_bind_* values, the experimental compatible partner distributions seem to be noisier than the docking score-based graphs. Comparing the normalized *P_bind_* values and the number of compatible partners for the best 1,000 compounds, a clear correlation can be detected (Figure S7). The steeper a given building block’s incline in the plot, the higher the effect of the building block position on ligand binding.

**Figure 8:**
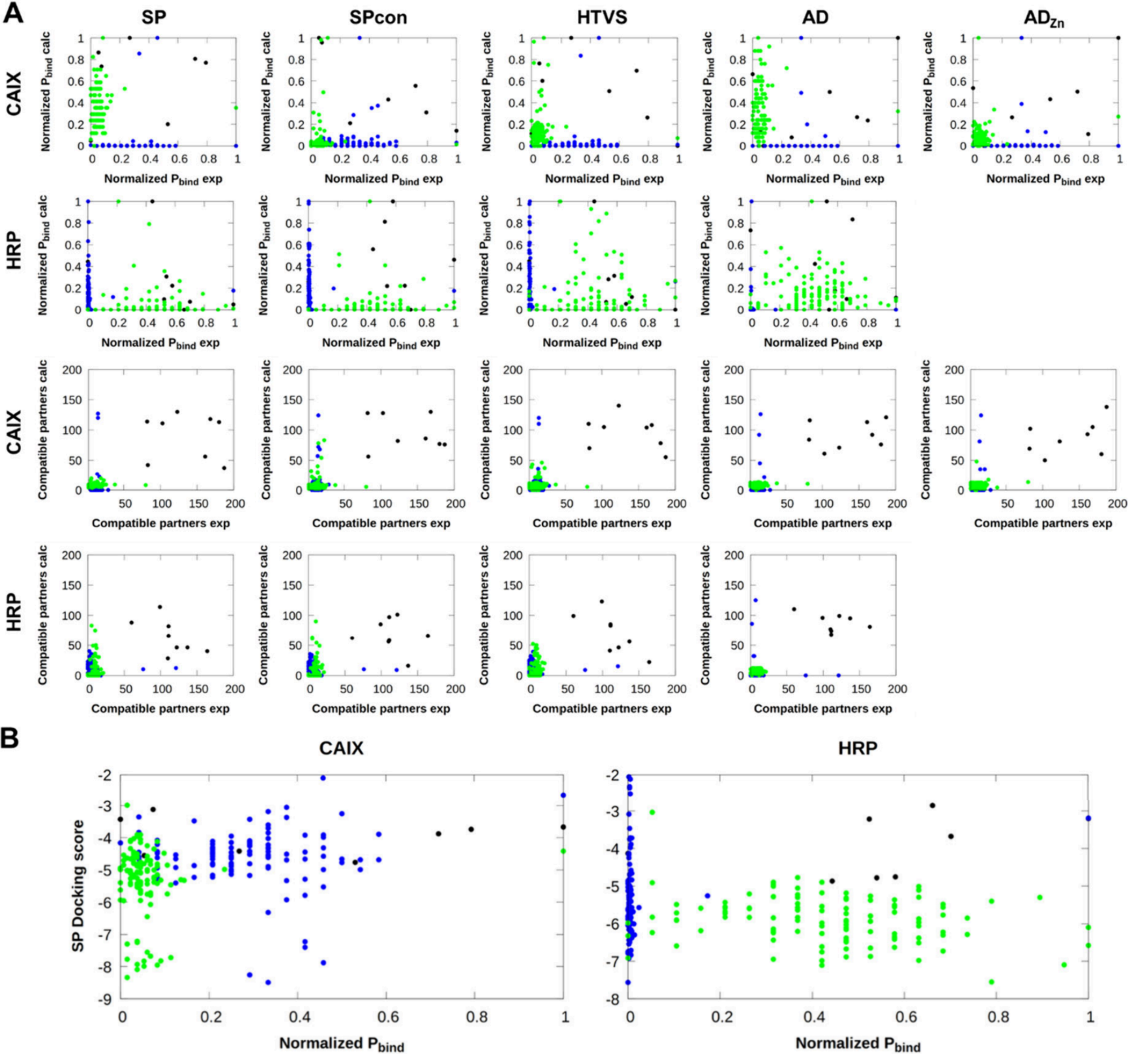
A) Comparison of the normalized *P_bind_* values and number of compatible partners against CAIX and HRP between the respective experimental hits vs the best 1,000 virtually screened compounds according to the docking algorithms SP, Spcon, HTVS, AD, and AD_Zn_. B) Relation between SP docking scores of individual building blocks and their respective normalized *P_bind_* values based on the experimental hits against CAIX and HRP (black: scaffold | blue: BB1 | green: BB2).

In the comparison of computed and experimental normalized *P_bind_* values and the number of compatible partners (Figure 8A), the given building blocks are scarcely marked as important by both experimental and computational evaluation (top right section of the plots). Our next aim was to study whether the docking of separate building blocks could recreate trends found in the experimental hits’ building blocks composition. We compared the SP docking scores of the various building blocks to their respective normalized *P_bind_* values (Figure 8B). We observed no clear correlation between the measures, even though one would expect lower (better) scored building blocks to possess higher normalized *P_bind_* values (bottom right section of the plots).

### Building Block Analysis – DEL-A Data Set

**Figure 9:**
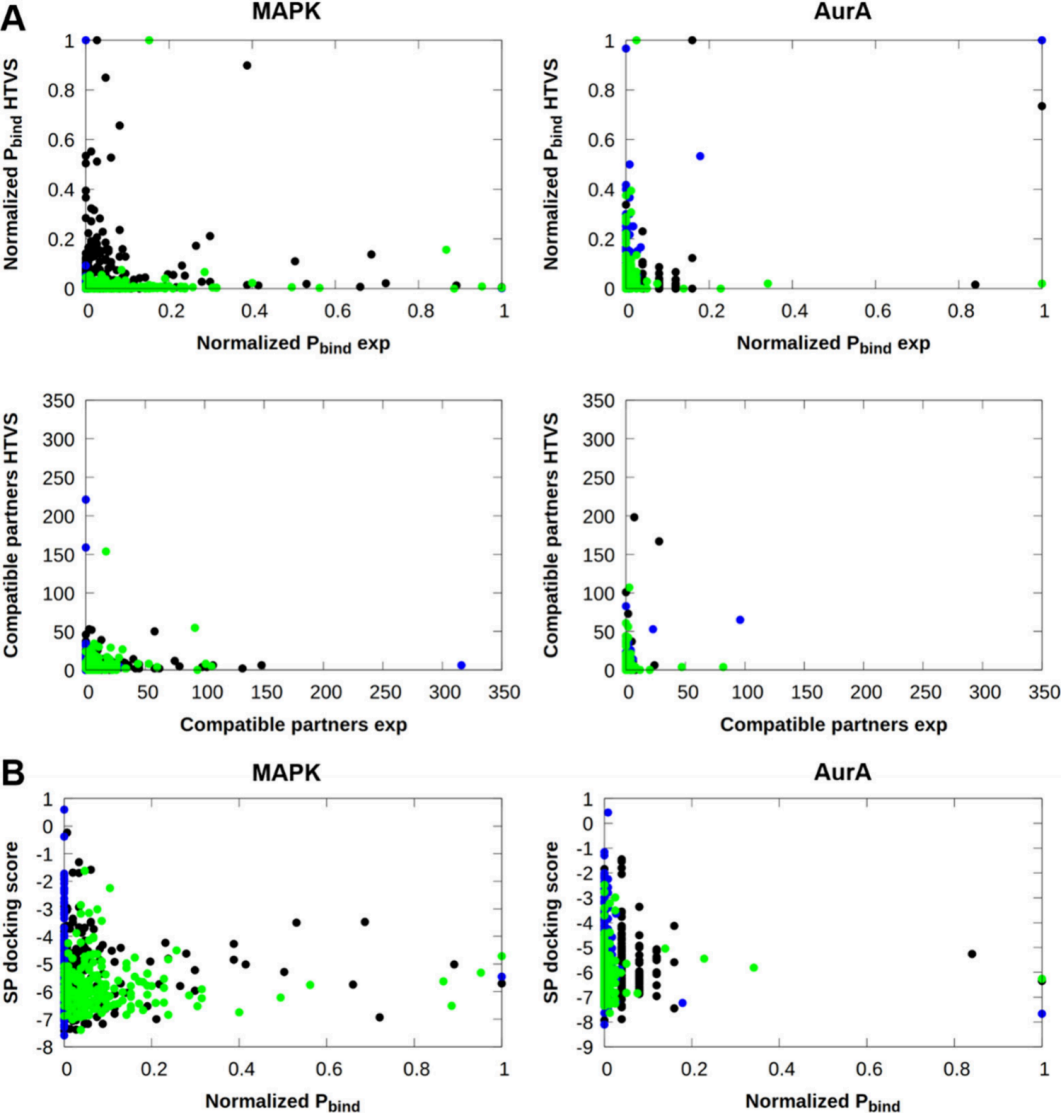
A) Comparison of the experimental hits’ and the best 1,000 virtually screened compounds’ (based on the HTVS docking scores) building block normalized *P_bind_* values and number of compatible partners against MAPK and AurA. B) Relation between the SP docking score of individual building blocks and their respective normalized *P_bind_* values based on the experimental hits against MAPK and AurA (black: BB1 | blue: BB2 | green: BB3).

A building block-based analysis on the experimental and the brute-force HTVS docking results of the DEL-A data set was also carried out. Normalized *P_bind_* and the number of compatible partners based on the building block composition of the hit compounds were calculated as described above. In Figure 9, comparison of the experimental and calculated *P_bind_* values and the number of compatible partners reveals no visible relation between the computational and experimental properties. The majority of the building blocks falls into the bottom left part of the plots, meaning that most of the building blocks hold low importance in hit classification. Building blocks possessing higher *P_bind_* values or a larger number of compatible partners among the experimental or HTVS hits are present mostly in the top left or bottom right sections of the plots, meaning that different building blocks were selected as essential molecular parts by the experiments vs virtual screening and the overlap between the building blocks with higher values (top right part of the plots) is scarce. This is an indication that the reproduction of the experimental ranking of building blocks is not feasible based on brute force-docking. For further results, including the individual normalized *P_bind_* values and the number of compatible partners, please refer to Figures S8–S12.

### Building Block Analysis – DEL-B Data Set

**Figure 10:**
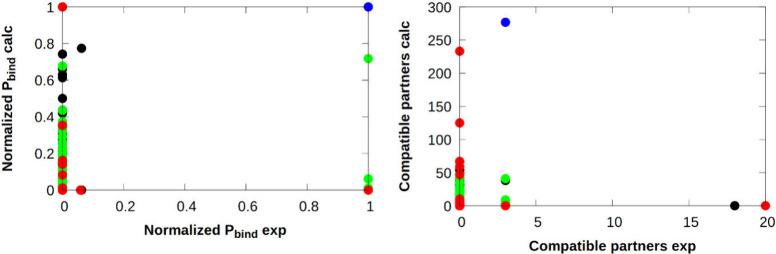
Comparison of the experimental hits’ and the best 1,000 virtually screened compounds’ (based on the Deep Docking VHL-values) building block normalized *P_bind_* values and the number of compatible partners against MAPK (black: BB1 | blue: BB2 | green: BB3 | red: BB4).

Analysis of the building block composition of experimental hits reveals that positions 1, 2, and 4 (BB1, BB2, BB4) are mostly utilized by the same set of fragments (Table S2). As the DEL-B hit set is composed of only 18 compounds among the library of hundreds of millions of molecules, the most we can realistically expect is for the computational methods to somewhat reproduce the occurrence of a few specific building blocks. Here, the most important building block (namely building block #**27** at the BB2 position, please refer to Figure S13), which is part of all the hit compounds, was successfully recovered computationally (top right point on the left panel of Figure 10); it was the most frequent building block among the best 1,000, 10,000, and 100,000 compounds as well, based on the VHL-values of the Deep Docking workflow applying Glide HTVS as the docking engine. It was found to be the only BB2 fragment among the best 1,000 compounds (Figure S13) and also possesses the highest number of compatible partners as well (Figure S14). Comparison of the computational *P_bind_* values and the number of compatible partners revealed that BB1 and BB3 positions hold similar importance, whereas BB4 and the previously discussed BB2 play more crucial roles in hit identification, with steeper inclinations on Figure S15. Plotting the single precision docking score of the individual fragment as a function of experimental normalized *P_bind_* values does not hold important information as the majority of the building blocks have a *P_bind_* value of 0 or 1 (Figure S16). However, BB2 #27 was among the top 10 compounds based on its SP docking score.

### Accessibility of the Linking Atoms – DEL-0, DEL-A, and DEL-B

A key limitation of evaluating the uHTVS protocols against DEL data sets is that docking does not account for the presence of the DNA tag, whose effect can be broken down to two factors. The first is the direct interaction of the DNA tag with the protein target. Here, the presence of a suitably long linker between the compound and the DNA tag ensures that on-target binding is achieved by the compound itself, while amplification or detection of the DNA tag in subsequent steps remains undisturbed. Thus, we can assume the direct effect of the DNA tag on binding to be negligible. The second factor is the orientation of the exit vector (i.e., the attachment point of the linker and the DNA tag) in the respective binding pose. This is potentially more problematic (and varies case-by-case), as the predicted binding pose might present the exit vector in a buried position within the binding site, making the respective binding pose physically unavailable for the full (DNA-tagged) compound.

To evaluate the ratio of the ligand poses that would be feasible for the actual DEL compound equipped with the DNA tag, we calculated an accessibility score of the exit vectors (linking atoms) at the respective docking poses of the DEL ligands. The accessibility score is defined as the repulsion potential between the examined atoms and the protein environment. Hence, an accessibility score of zero means accessible link atoms and a feasible pose for the compound of the given DEL. Table 6 shows the ratio of feasible poses compared to the complete set of poses. Generally, around half of the resulting docking poses is in fact feasible for the full compounds, while the remaining poses are favored by the given docking algorithm for the untagged DEL compound (but these cannot reproduce experimental results). As anticipated, a higher ratio of feasible poses can be achieved by biasing the docking algorithm with positional constraints (Spcon vs SP). Interestingly, random sampling of the DEL-B library also resulted in similar ratios of feasible poses, almost identical with the ratio of the best 100,000 prospective hits of the Deep Docking assisted uHTVS workflow.

**Table 6:** Ratios of Feasible Poses (Accessibility Score = 0.0) for the Various Sets of Molecules

Data set	Docking method	Examined compounds	Target	Ratio of feasible poses (%)
DEL-0	Glide SP	all	CAIX	47.63
DEL-0	Glide Spcon	all	CAIX	57.86
DEL-0	Glide HTVS	all	CAIX	51.42
DEL-0	AD	all	CAIX	32.21
DEL-0	AD_Zn_	all	CAIX	33.47
DEL-0	Glide SP	all	HRP	55.24
DEL-0	Glide Spcon	all	HRP	61.83
DEL-0	Glide HTVS	all	HRP	55.82
DEL-0	AD	all	HRP	54.43
DEL-A	Glide HTVS	best 100,000	MAPK	32.26
DEL-A	Glide HTVS + DD	best 100,000	MAPK	52.61
DEL-A	AD + DD	all prospective hits	MAPK	23.43
DEL-A	Glide HTVS	best 100,000	AurA	45.98
DEL-A	Glide HTVS + DD	best 100,000	AurA	41.32
DEL-A	AD + DD	all prospective hits	AurA	35.41
DEL-B	Glide HTVS + DD	best 100,000	MAPK	58.66
DEL-B	Glide HTVS	random 1,000	MAPK	56.44
DEL-B	Glide HTVS	random 10,000	MAPK	57.35
DEL-B	Glide HTVS	random 100,000	MAPK	57.56

Finally, we examined if filtering out unfeasible poses would improve the ROC curves of the DEL-0 data set, hence, the reproduction of experimental results. Interestingly, the results have not been improved; in fact, the pAUC values are close to random sampling (Figure S17 and Table S3). This suggests that pose invalidity is not the main issue causing the discrepancy between the experimental and calculated results.

## Conclusion

A comparative study on ultrahigh throughput virtual screening (uHTVS) vs experimental DNA-encoded library (DEL) screening was carried out. Various docking tools, including AI-enabled workflows, were utilized to find the most promising strategy for exploring ultralarge chemical spaces, represented in our case studies by DEL screening sets with experimental information on all compounds (actives and inactives). Three DEL sets were selected with sizes that cover multiple orders of magnitude. This enabled us to compare the brute-force approach and a machine-learning (ML) based workflow as well. Among the brute-force docking studies, AutoDockGPU was found to underperform Glide (single precision and HTVS). In general, the docking of all DEL compounds revealed only slightly better ROC curves than a random selection of molecules. More interestingly, the initial steep incline of the ROC curves was missing completely for the DEL-A data set; hence, the early enrichment factor values were found to be near 1. For the larger DEL-B data set, EF values of 11.1 at 1% and 5.56 at 2% were detected. In the case of the DEL-A data set, where both the brute-force and AI-assisted approaches were applied, the all-ligand approach seemed to outperform the machine-learning model. This is not surprising, as the ML model was trained to reproduce the computational docking results; hence, a better performance would have been unexpected. The overall performance of the AI-enabled workflow was found to be highly dependent on the docking engine and target protein, which is also supported by the docking score distributions. Deep Docking combined with AutoDockGPU showed very poor results in terms of experimental hit finding and docking score distributions, whereas the Glide-HTVS based ML model performed reasonably well on AurA but somewhat poorly on MAPK. For the largest data set, the Deep Docking-Glide HTVS workflow with a VHL-value ≥ 0.95 filter was not able to find any experimental hits; however, without applying the filter (i.e., taking all 150 million prospective virtual hits into consideration), 77.8% of the experimental hits were identified. These results suggest that Deep Docking works well with ultralarge (over 100 million compounds) databases, and filtering all prospective virtual hits based on VHL-values should be avoided. However, the amount of prospective virtual hits is unfeasibly large for standard docking, and further filtering is still necessary to process the results. The building block-based analysis of the data sets revealed no unambiguous relation between experimental and computational results; however, in the case of the DEL-B study, the experimentally most frequent building block was also found to be the most recurring one among the computational hits.

The modest performance of the computational methods on these data sets can be attributed, at least partly, to the noisy nature of the experimental on-DNA hits. Specifically, in the case of the first DEL data set, recovery of the off-DNA hits showed much better ROC curves, which suggests that the computational tools are more suitable to recover the more thoroughly validated off-DNA hits. Another possible issue is the feasibility of the resultant poses of the docking algorithms. By default, the docking procedure does not account for the DNA tag (or the linker), meaning that the resulting binding pose might present the exit vector (i.e., the attachment point of the linker and DNA tag) in a buried position within the binding site, making the respective binding pose physically unavailable for the full (DNA-tagged) compound. These aspects may influence the observed performance of uHTVS workflows negatively when evaluated against DEL data sets, although we have shown here that this is not the main reason for the observed poor performances.

As for the specific case of DEL libraries, experimental DEL screening is not replaceable with simple computational docking tools at the moment. However, as uHTVS workflows can be performed even on standard computer infrastructures in a sensible time, they can serve as valuable complementary tools to further investigate or improve the wet-lab results or provide an additional layer for off-DNA compound selection by docking the best results of the DEL screen or by docking the whole data set, acquiring information about which molecules (or which molecules containing specific building blocks) are worth synthesizing off-DNA.

More generally, a clear advantage of uHTVS approaches is the wider chemical space that is accessible simultaneously. DEL compounds contain a limited number of building blocks (typically 3–4) attached with one type of reaction per building block: for example, the DEL-A and DEL-B data sets referenced in this work contain (192+192+192) and (192+32+340+384) building blocks in the respective attachment positions. By contrast, there is practically no limitation to the chemistries considered when compiling make-on-demand virtual libraries, e.g. the Enamine REAL space is “assembled via more than 167 well-validated parallel synthesis protocols applied to over 143 000 qualified reagents and building blocks”.^55^ Thus, uHTVS currently remains the only feasible way to explore chemical spaces that cannot be covered by a single DEL library.

## Data Availability

DEL data sets are available at 10.1021/jacs.9b01203 (DEL-0) and 10.1038/nchembio.211 (DEL-A and DEL-B). Docking results and source data of the figures are deposited at 10.5281/zenodo.13221737. Scripts used for the Deep Docking workflow are available at https://github.com/keserulab/uHTVS_toolkit/.

## Supporting Information Available

Supplementary tables with additional information regarding the off-DNA hits of DEL-0 (Table S1), the experimental hits of DEL-B (Table S2) and the pAUC values of the DEL-0 dockings after filtering out infeasible poses (Table S3). Supplementary figures including the description of the accessibility calculations (Figure S1); additional ROC curves (Figures S2, S17); plots of the building block distributions and compositions of various subsets of the complete libraries based on the docking scores, normalized *P_bind_* values, and the number of compatible partners (Figures S3, S5–S6, S8–S11, S13–S14); graphs of virtual-hit-likeness score distributions (Figure S4); scatterplots comparing the normalized *P_bind_* values and the number of compatible partners of specific building blocks (Figures S7, S12, S15); comparison of the experimental normalized *P_bind_* and the SP docking score of DEL-B building blocks (Figure S16); Deep Docking statistics (Figure S18). Supplementary notes on the benchmarking statistics of the Deep Docking workflows (PDF)
