# Supplementary material for: Exploring Chemical Spaces in the Billion Range: Is Docking a Computational Alternative to DNA-Encoded Libraries?
Source: J Chem Inf Model. 2024 Sep 21;64(23):8963–79. doi: 10.1021/acs.jcim.4c00803 (PMC11632764; doi:10.1021/acs.jcim.4c00803)
Supplement: Supplementary file 1 — ci4c00803_si_001.pdf [file ci4c00803_si_001.pdf]

Supplementary information

Exploring chemical spaces in the billion range: is docking a computational alternative to DNA-encoded libraries?

Levente M. Mihalovits[1], Tibor V. Szalai[1,2], Dávid Bajusz[1], György M. Keserű[1,3]*

[1]*Medicinal Chemistry Research Group and Drug Innovation Centre, HUN-REN Research Centre for Natural Sciences, Magyar tudósok krt. 2, 1117 Budapest, Hungary*

[2]*Department of Inorganic and Analytical Chemistry, Faculty of Chemical Technology and Biotechnology, Budapest University of Technology and Economics, Műegyetem rkp. 3., H-1111 Budapest, Hungary*

[3] *Department of Organic Chemistry and Technology, Faculty of Chemical Technology and Biotechnology Budapest University of Technology and Economics Műegyetem rkp. 3., H-1111 Budapest, Hungary*

*Corresponding author: György M. Keserű, keseru.gyorgy@ttk.hu

Table of content

Supplementary tables 2

Supplementary figures 4

Supplementary notes 27

Supplementary tables

Table S1. Compounds that were evaluated against CAIX by off DNA synthesis

id	Structure	cpd_id	Scaffold	BB1	BB2	IC_{50} (nM)
**56218**	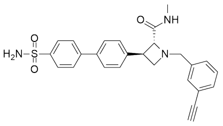	**5a**	4	9	22	24.3
**10450**	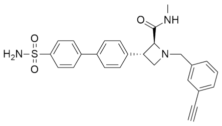	**5b**	3	9	22	25.6
**56336**	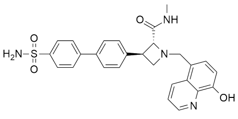	**6a**	4	99	22	23.7
**10568**	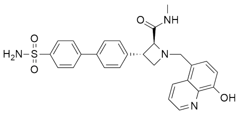	**6b**	3	99	22	20.1
**60112**	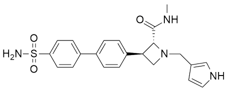	**7a**	4	41	22	20.8
**14344**	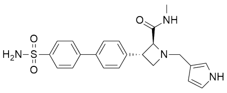	**7b**	3	41	22	68.7
**56258**	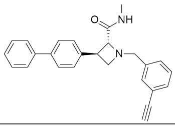	**10a**	4	9	37	>100000
**10490**	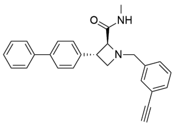	**10b**	3	9	37	>100000

Table S2. DEL-B experimental hits' building block composition

Serial number	Compound ID	BB1 ID	BB2 ID	BB3 ID	BB4 ID
**1**	L648745773	30	27	9	74
**2**	L648966381	30	27	12	74
**3**	L649701741	30	27	22	74
**4**	L651760749	30	27	50	74
**5**	L652716717	30	27	63	74
**6**	L653525613	30	27	74	74
**7**	L653966829	30	27	80	74
**8**	L655775668	145	27	104	4
**9**	L656467053	30	27	114	74
**10**	L656553994	65	27	115	74
**11**	L657496557	30	27	128	74
**12**	L658158381	30	27	137	74
**13**	L659776173	30	27	159	74
**14**	L660143853	30	27	164	74
**15**	L661614573	30	27	184	74
**16**	L666173805	30	27	246	74
**17**	L667865133	30	27	269	74
**18**	L668968173	30	27	284	74

Table S3. Docking results of the DEL-0 dataset after filtering out infeasible docking poses. For the ROC curves please refer to **Figure S17.**

Library	Target	Docking method	pAUC
**DEL-0**	CAIX	SP	0.532
**DEL-0**	CAIX	SPcon	0.539
**DEL-0**	CAIX	HTVS	0.533
**DEL-0**	CAIX	AD	0.512
**DEL-0**	CAIX	AD_{Zn}	0.501
**DEL-0**	HRP	SP	0.559
**DEL-0**	HRP	SPcon	0.549
**DEL-0**	HRP	HTVS	0.561
**DEL-0**	HRP	AD	0.481

Supplementary figures

Figure S1. *Atoms whose accessibility score was evaluated for DEL pose feasibility.*

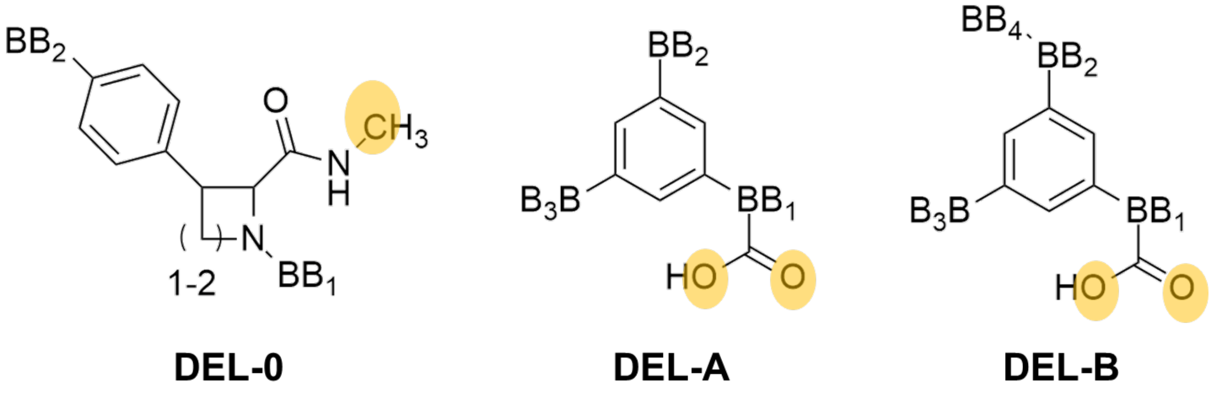

Figure S2. *ROC curves of the virtual screening methods using the DEL-0 dataset.*

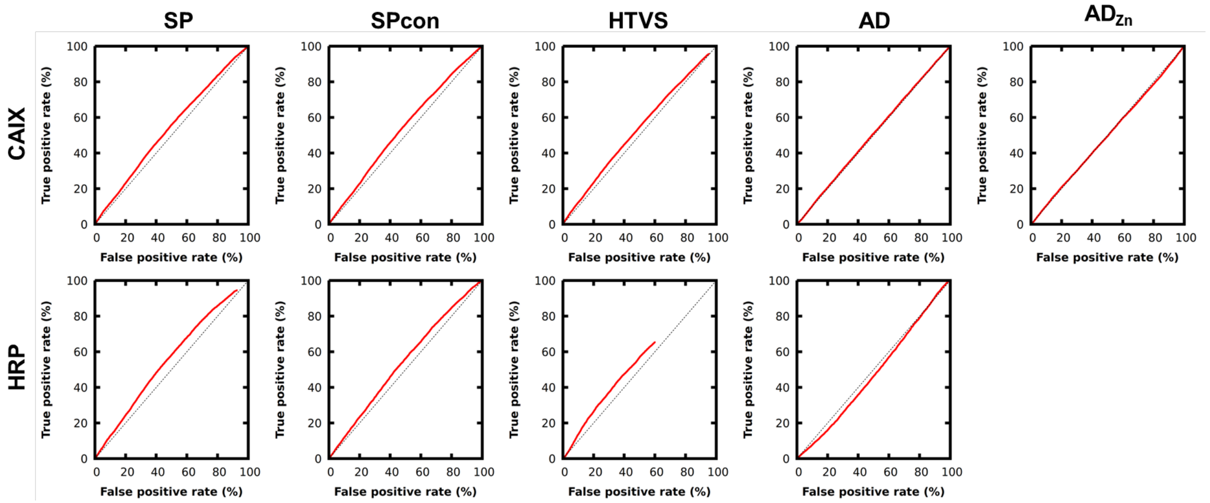

Figure S3. *Building block composition of compounds possessing docking scores around the distribution peaks of the SP (A), SPcon (B) and HTVS (C) datasets of CAIX. Distributions belonging to the lower and higher peaks are shown with blue and orange, respectively. The applied docking score filters are the following: SP (-7.5<lower<-6.5, -4.0<higher<-3.0), SPcon (-8.0<lower<-7.0, -4.1<higher<-3.1), HTVS (-6.6<lower<-5.6, -3.3<higher<-2.3)*

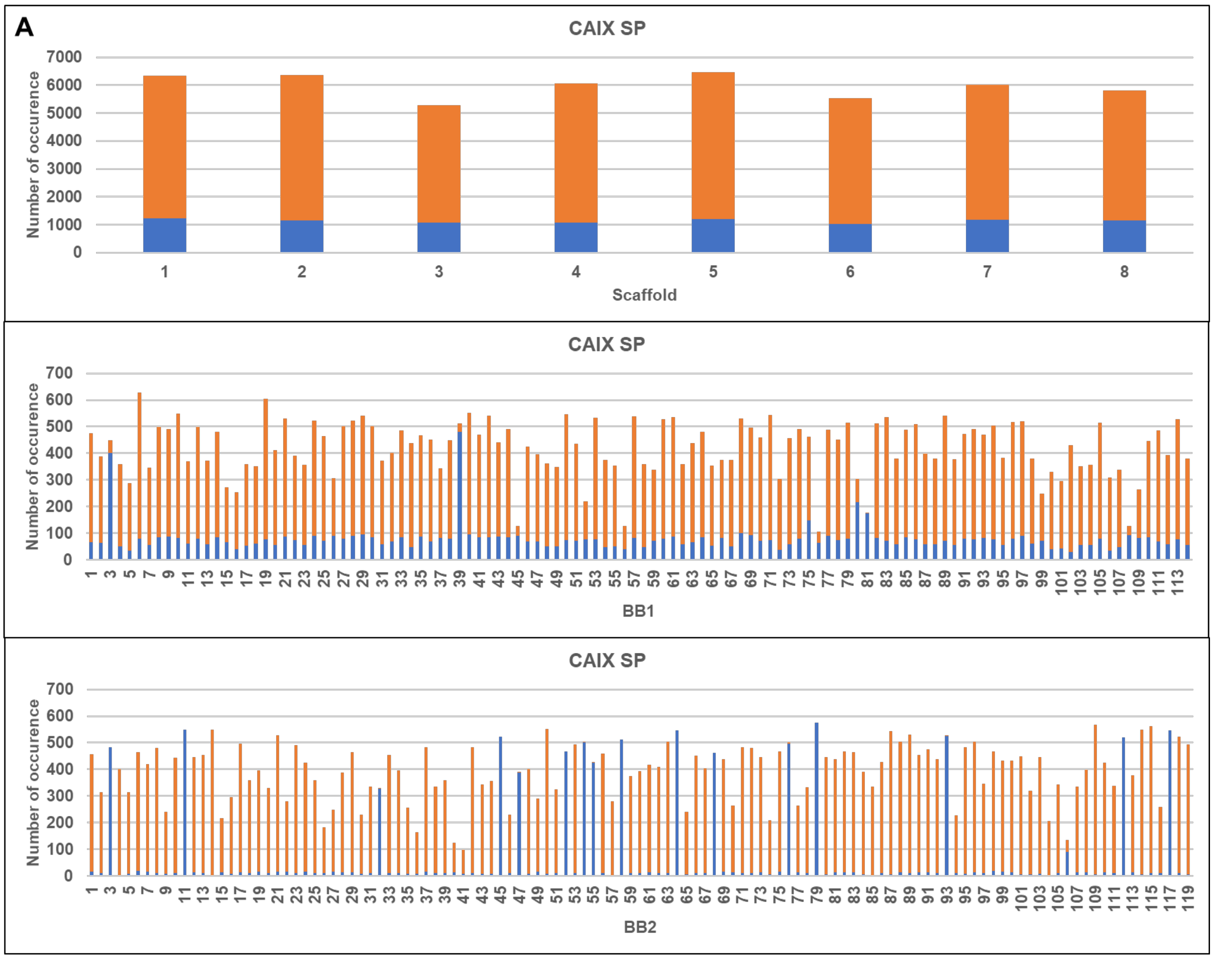

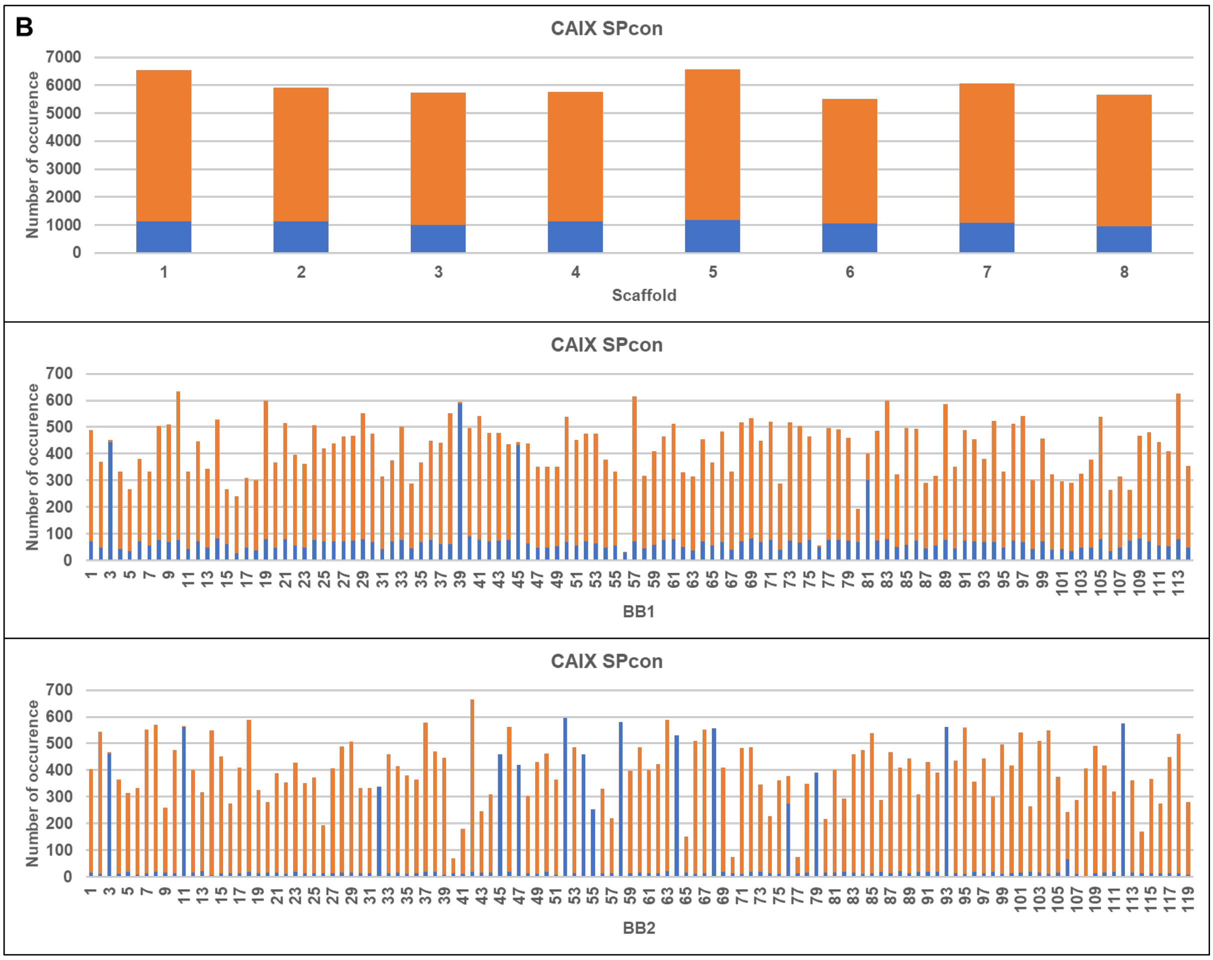

B
CAIX SPcon
Number of occurence
Scaffold
CAIX SPcon
Number of occurence
BB1
CAIX SPcon
Number of occurence
BB2

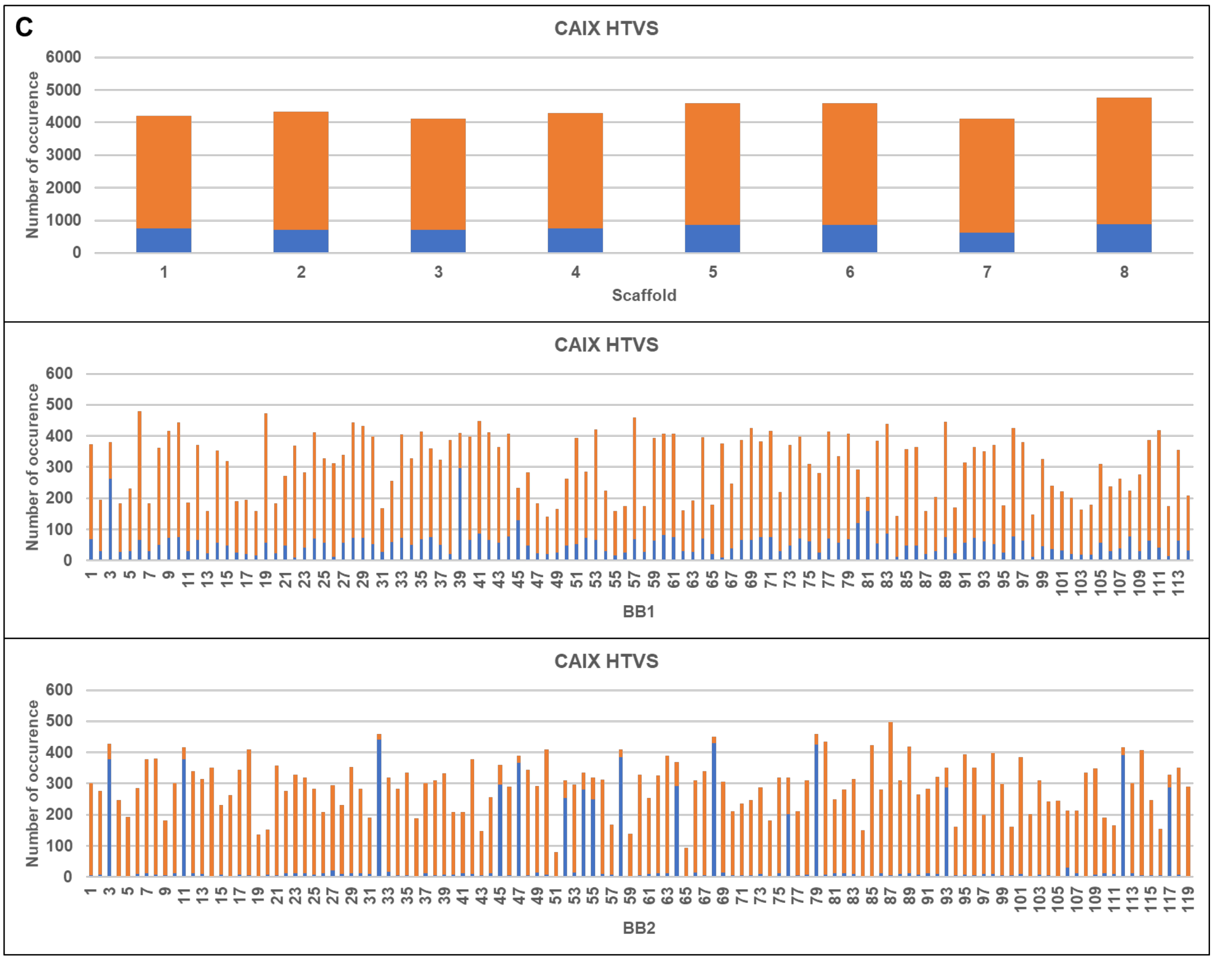
C
CAIX HTVS
Number of occurence
Scaffold
CAIX HTVS
Number of occurence
BB1
CAIX HTVS
Number of occurence
BB2

Figure S4. *VHL-value distributions (top: linear scale, bottom: logarithmic scale) for the virtual hits predicted by the DeepDocking workflow. (A) DEL-A dataset against AurA with AutoDockGPU (B) DEL-A dataset against AurA with Glide HTVS (C) DEL-A dataset against MAPK with AutoDockGPU (D) DEL-A dataset against MAPK with Glide HTVS (E) DEL-B dataset against MAPK with Glide HTVS*

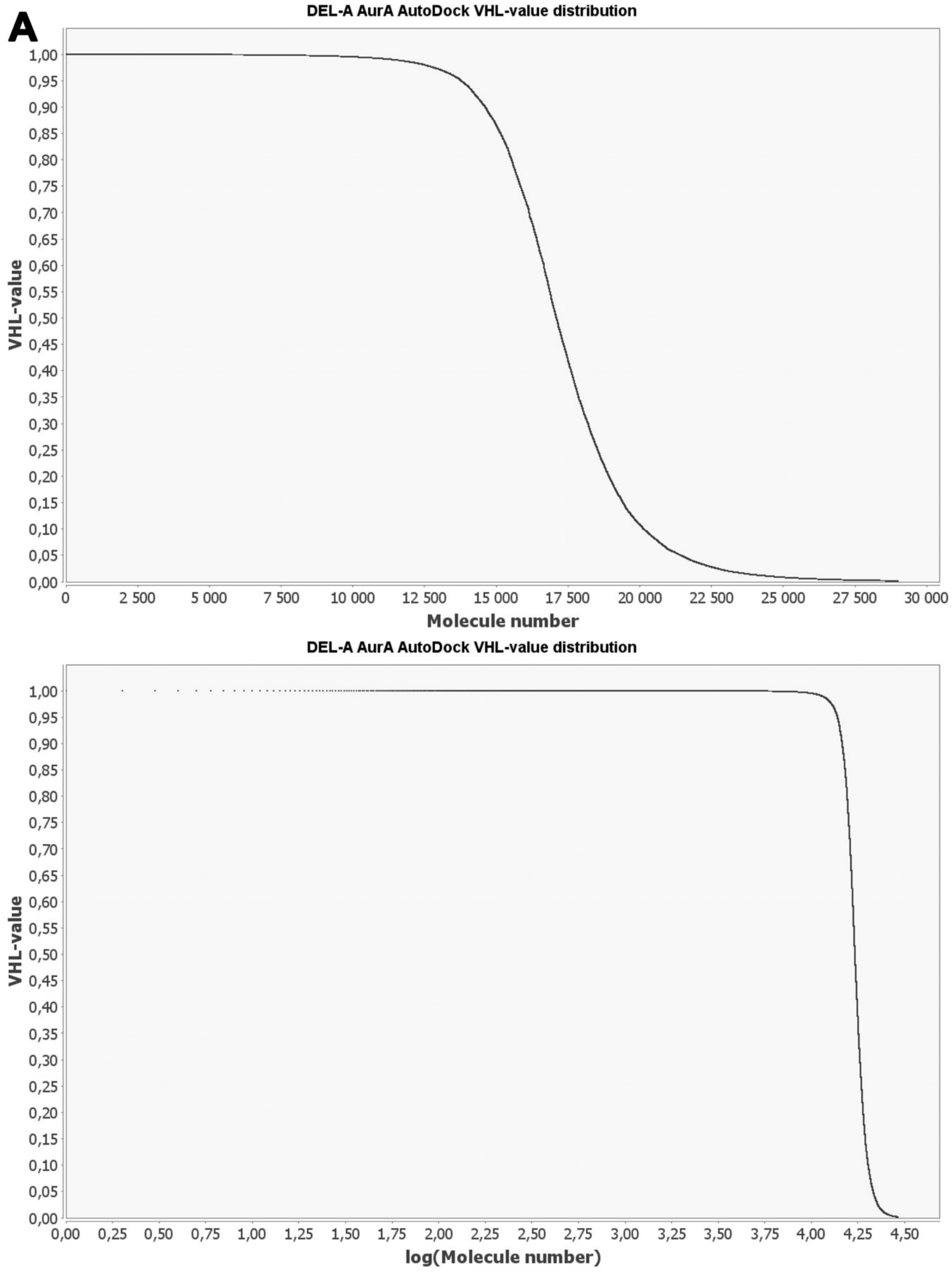

B

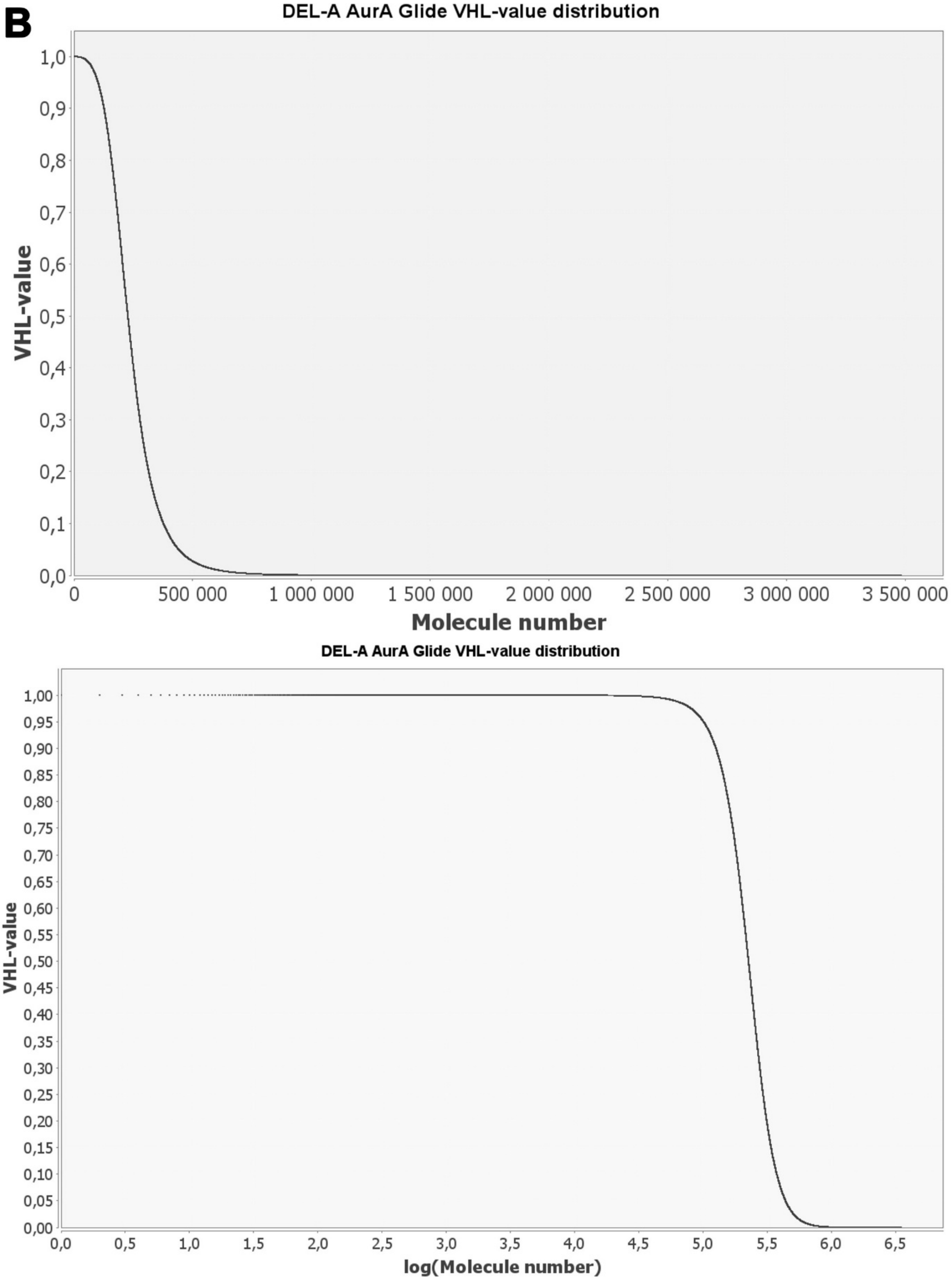
DEL-A AurA Glide VHL-value distribution
VHL-value
1,0
0,9
0,8
0,7
0,6
0,5
0,4
0,3
0,2
0,1
0,0
0
500 000
1 000 000
1 500 000
2 000 000
2 500 000
3 000 000
3 500 000
Molecule number
DEL-A AurA Glide VHL-value distribution
VHL-value
1,00
0,95
0,90
0,85
0,80
0,75
0,70
0,65
0,60
0,55
0,50
0,45
0,40
0,35
0,30
0,25
0,20
0,15
0,10
0,05
0,00
0,0
0,5
1,0
1,5
2,0
2,5
3,0
3,5
4,0
4,5
5,0
5,5
6,0
6,5
log(Molecule number)

C

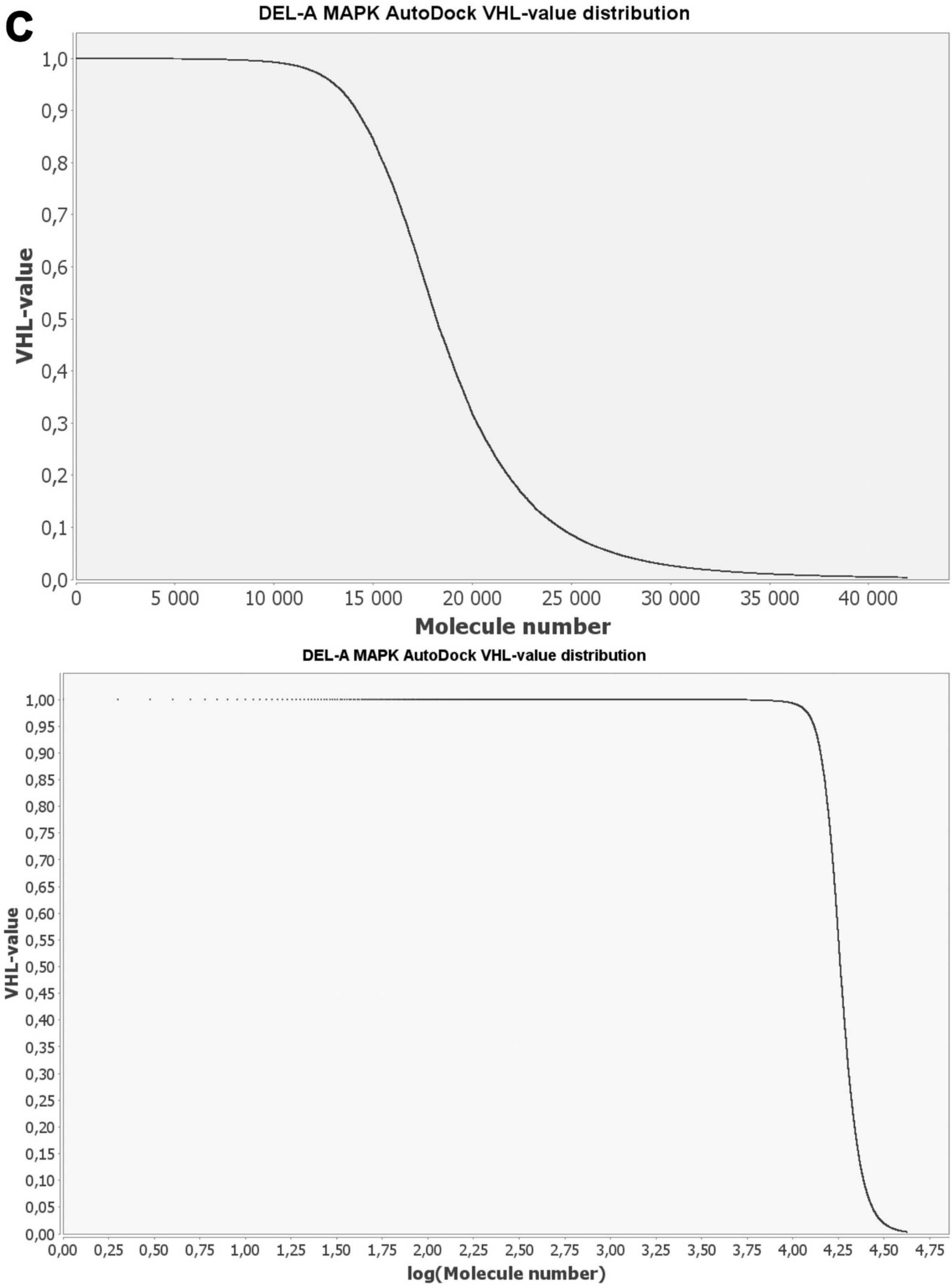

DEL-A MAPK AutoDock VHL-value distribution
VHL-value
Molecule number
DEL-A MAPK AutoDock VHL-value distribution
VHL-value
log(Molecule number)

D

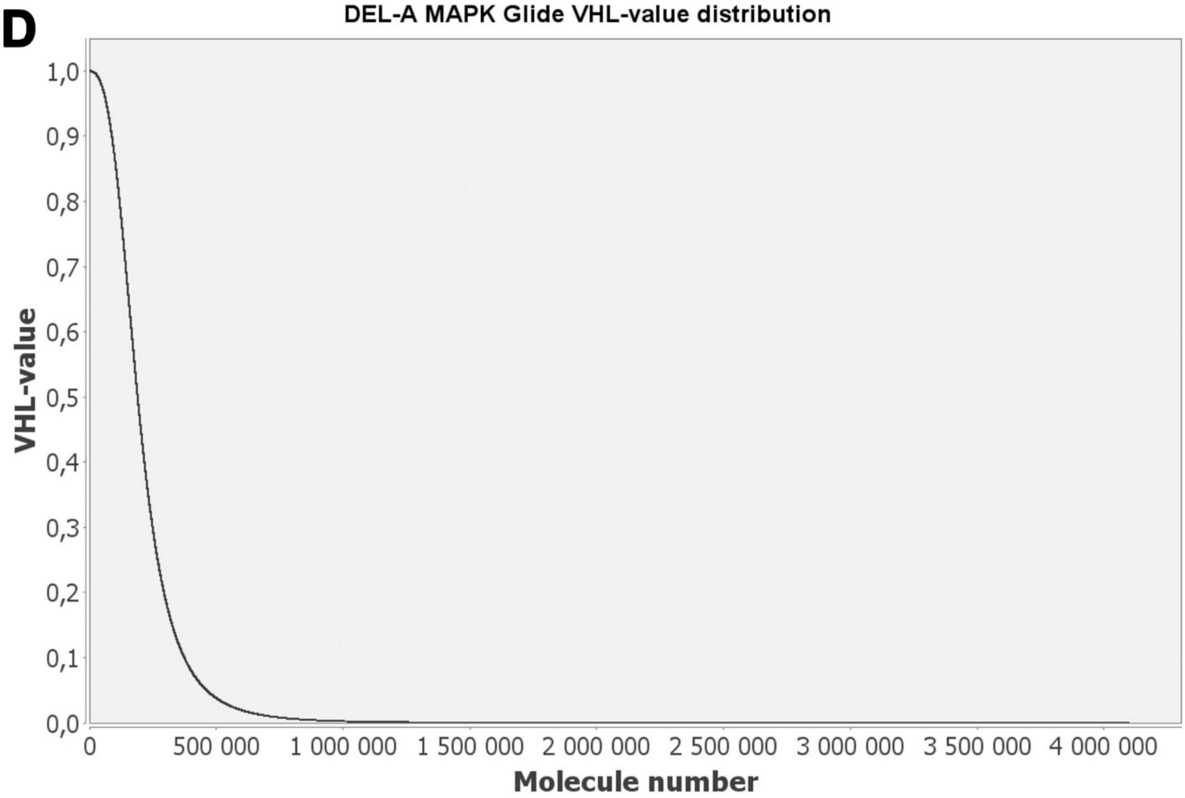
DEL-A MAPK Glide VHL-value distribution
VHL-value
1,0
0,9
0,8
0,7
0,6
0,5
0,4
0,3
0,2
0,1
0,0
0
500 000
1 000 000
1 500 000
2 000 000
2 500 000
3 000 000
3 500 000
4 000 000
Molecule number

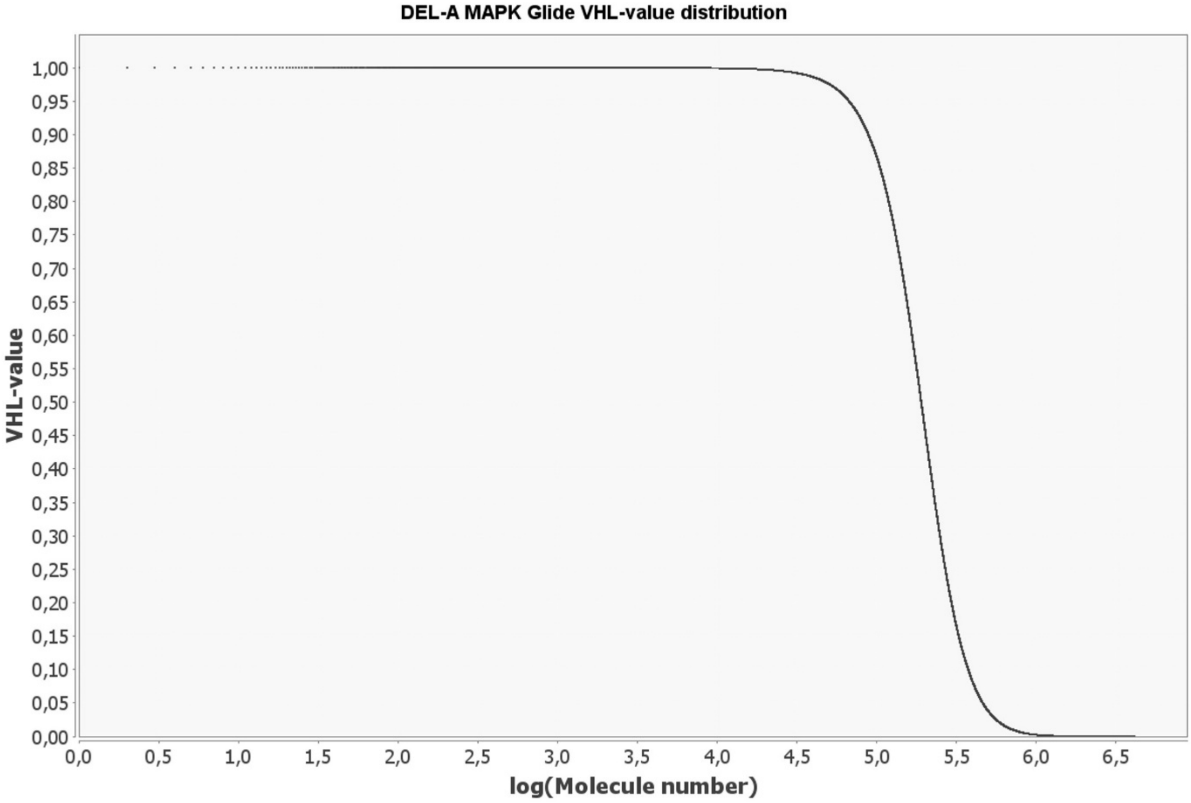
DEL-A MAPK Glide VHL-value distribution
VHL-value
1,00
0,95
0,90
0,85
0,80
0,75
0,70
0,65
0,60
0,55
0,50
0,45
0,40
0,35
0,30
0,25
0,20
0,15
0,10
0,05
0,00
0,0
0,5
1,0
1,5
2,0
2,5
3,0
3,5
4,0
4,5
5,0
5,5
6,0
6,5
log(Molecule number)

E

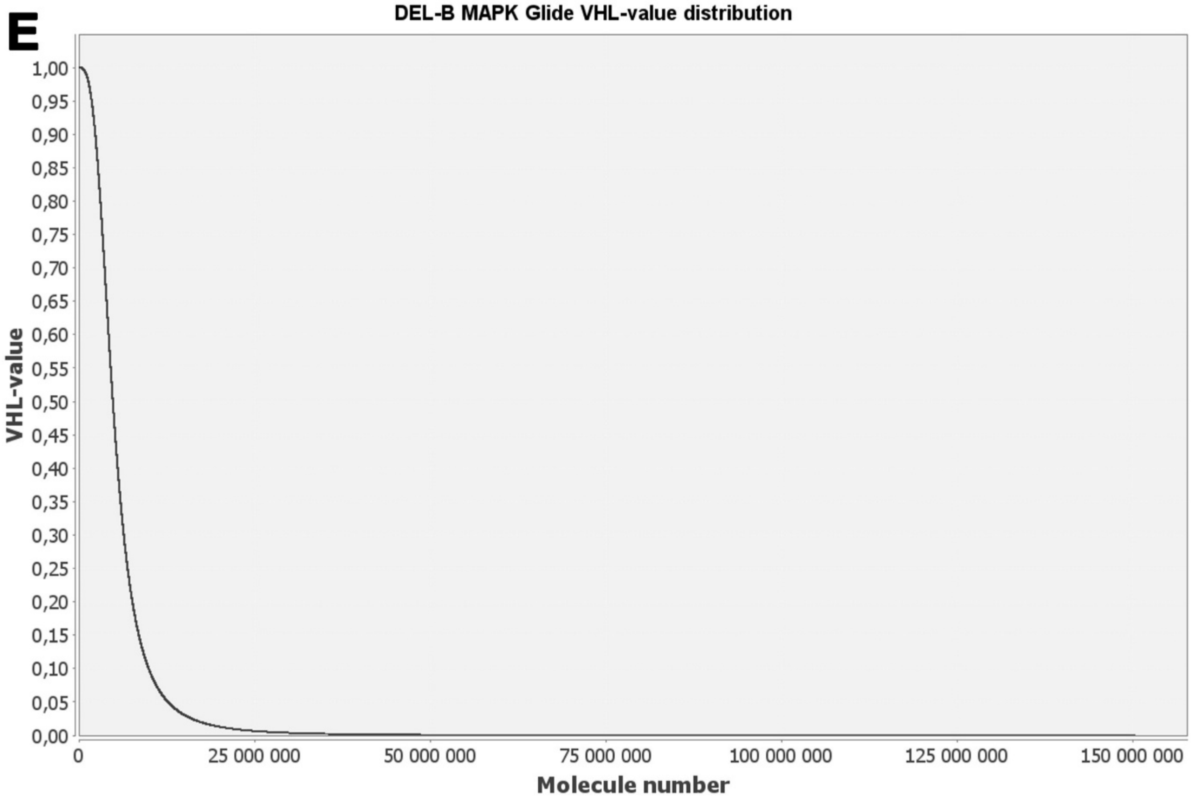

DEL-B MAPK Glide VHL-value distribution
VHL-value
1,00
0,95
0,90
0,85
0,80
0,75
0,70
0,65
0,60
0,55
0,50
0,45
0,40
0,35
0,30
0,25
0,20
0,15
0,10
0,05
0,00
0
25 000 000
50 000 000
75 000 000
100 000 000
125 000 000
150 000 000
Molecule number

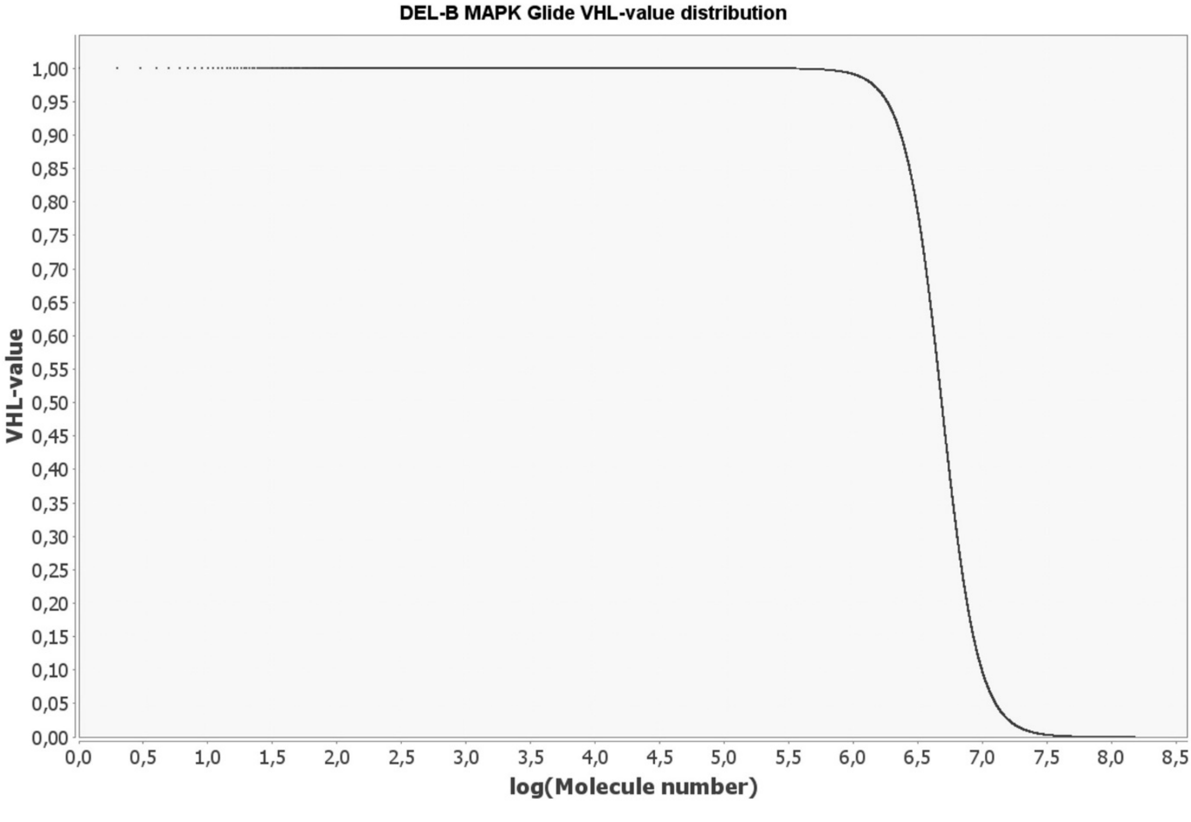

DEL-B MAPK Glide VHL-value distribution
VHL-value
1,00
0,95
0,90
0,85
0,80
0,75
0,70
0,65
0,60
0,55
0,50
0,45
0,40
0,35
0,30
0,25
0,20
0,15
0,10
0,05
0,00
0,0
0,5
1,0
1,5
2,0
2,5
3,0
3,5
4,0
4,5
5,0
5,5
6,0
6,5
7,0
7,5
8,0
8,5
log(Molecule number)

Figure S5. *Normalized P_{bind} values of the various building blocks at specific positions using the best 100 (black), 1000 (blue) and 13020 (green) compounds based on the SP, SPcon, HTVS, AD, AD_{Zn} docking scores (first five or four columns) and Fn values (last column) measured against CAIX (panel A-C) and HRP (panel D-F).*

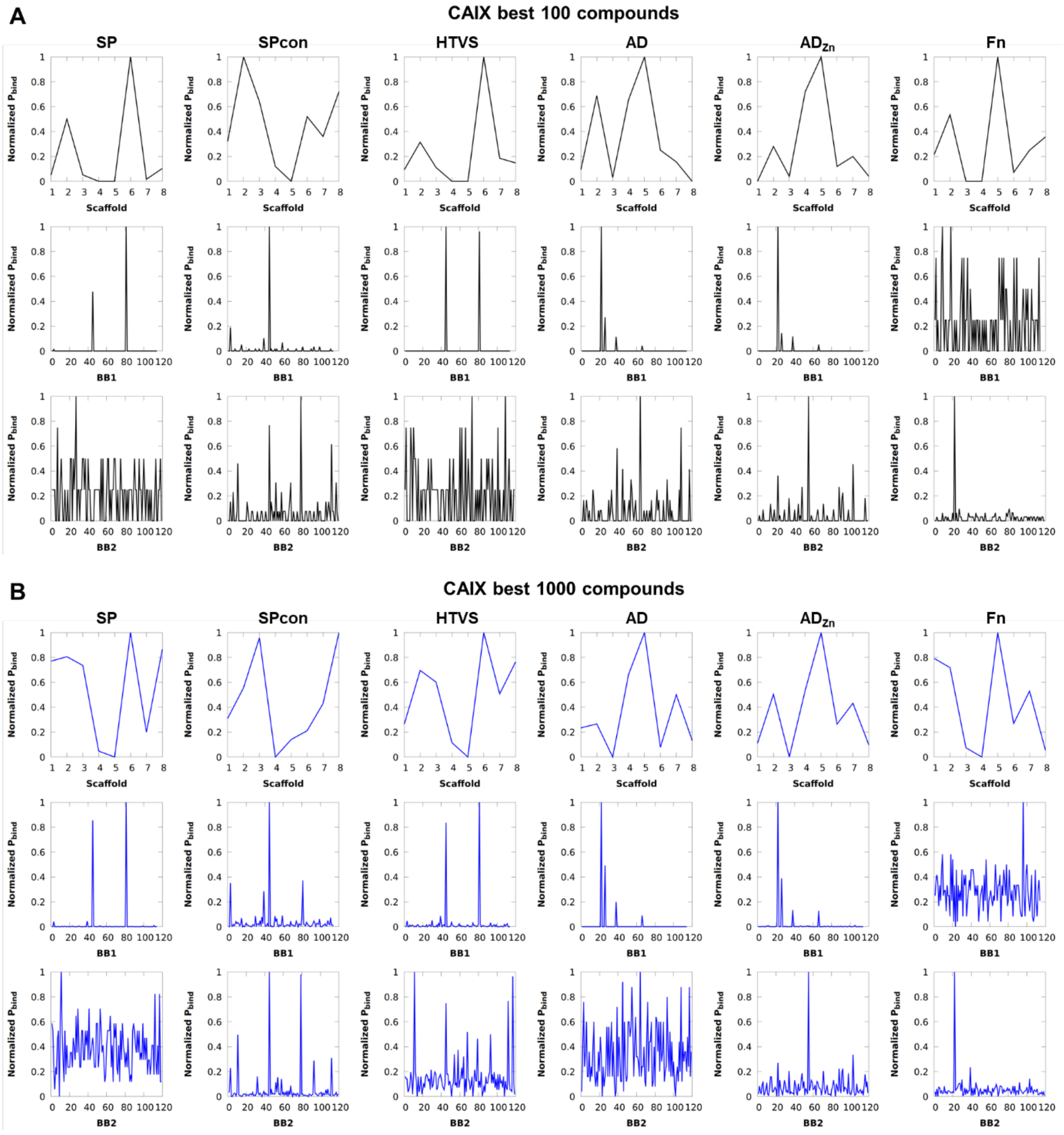

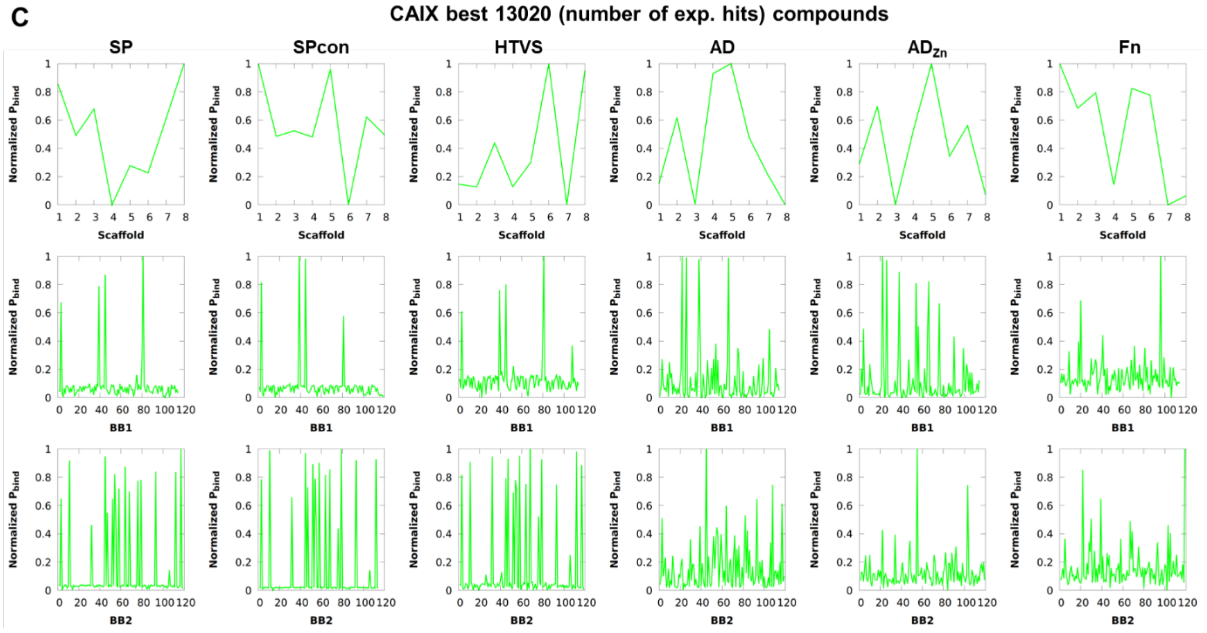
C
CAIX best 13020 (number of exp. hits) compounds
SP
SPcon
HTVS
AD
AD$_{Zn}$
Fn
Normalized P_{bind}
Scaffold
BB1
BB2

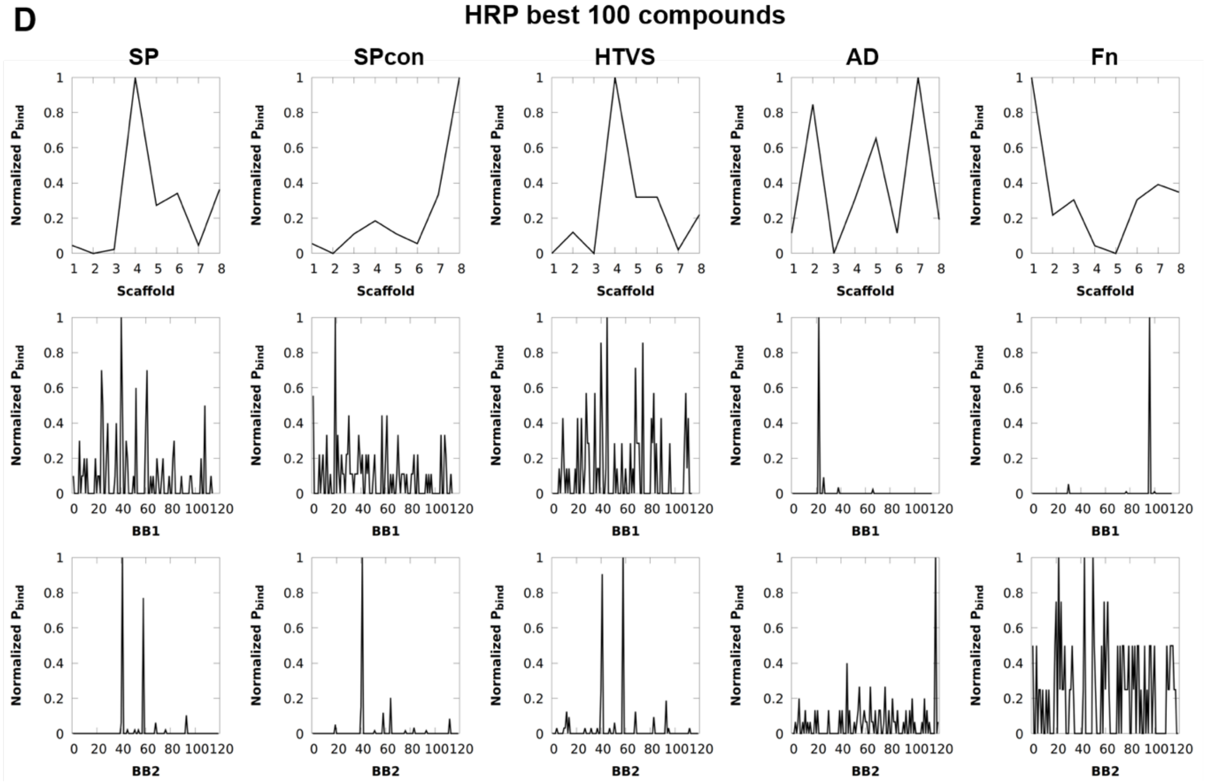
D
HRP best 100 compounds
SP
SPcon
HTVS
AD
Fn
Normalized P_{bind}
Scaffold
BB1
BB2

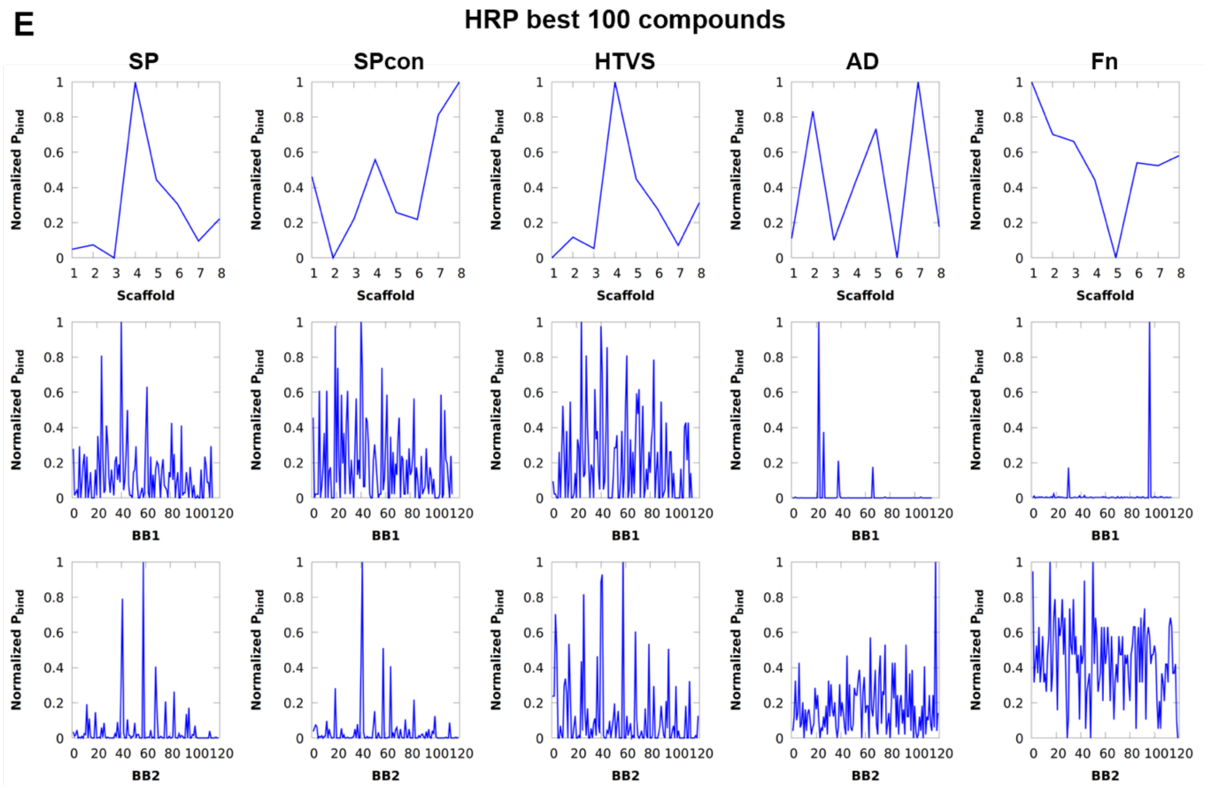
E
HRP best 100 compounds
SP
SPcon
HTVS
AD
Fn
Normalized Pbind
Scaffold
BB1
BB2

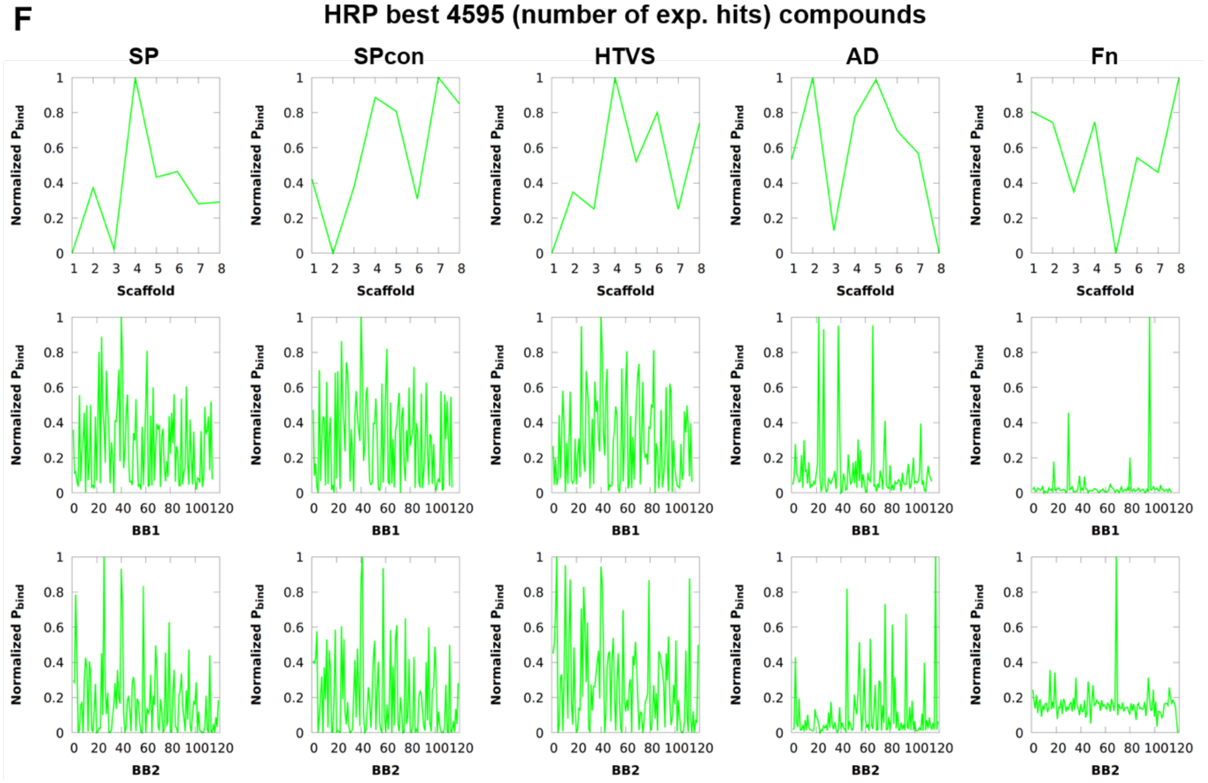
F
HRP best 4595 (number of exp. hits) compounds
SP
SPcon
HTVS
AD
Fn
Normalized Pbind
Scaffold
BB1
BB2

Figure S6. *Number of compatible partners of the various building blocks at specific positions using the best 100 (black), 1000 (blue) and 13020 or 4595 (green) compounds based on the SP, SPcon, HTVS, AD, AD_{Zn} docking scores (first five or four columns) and Fn values (last column) measured against CAIX (panel A-C) and HRP (panel D-F).*

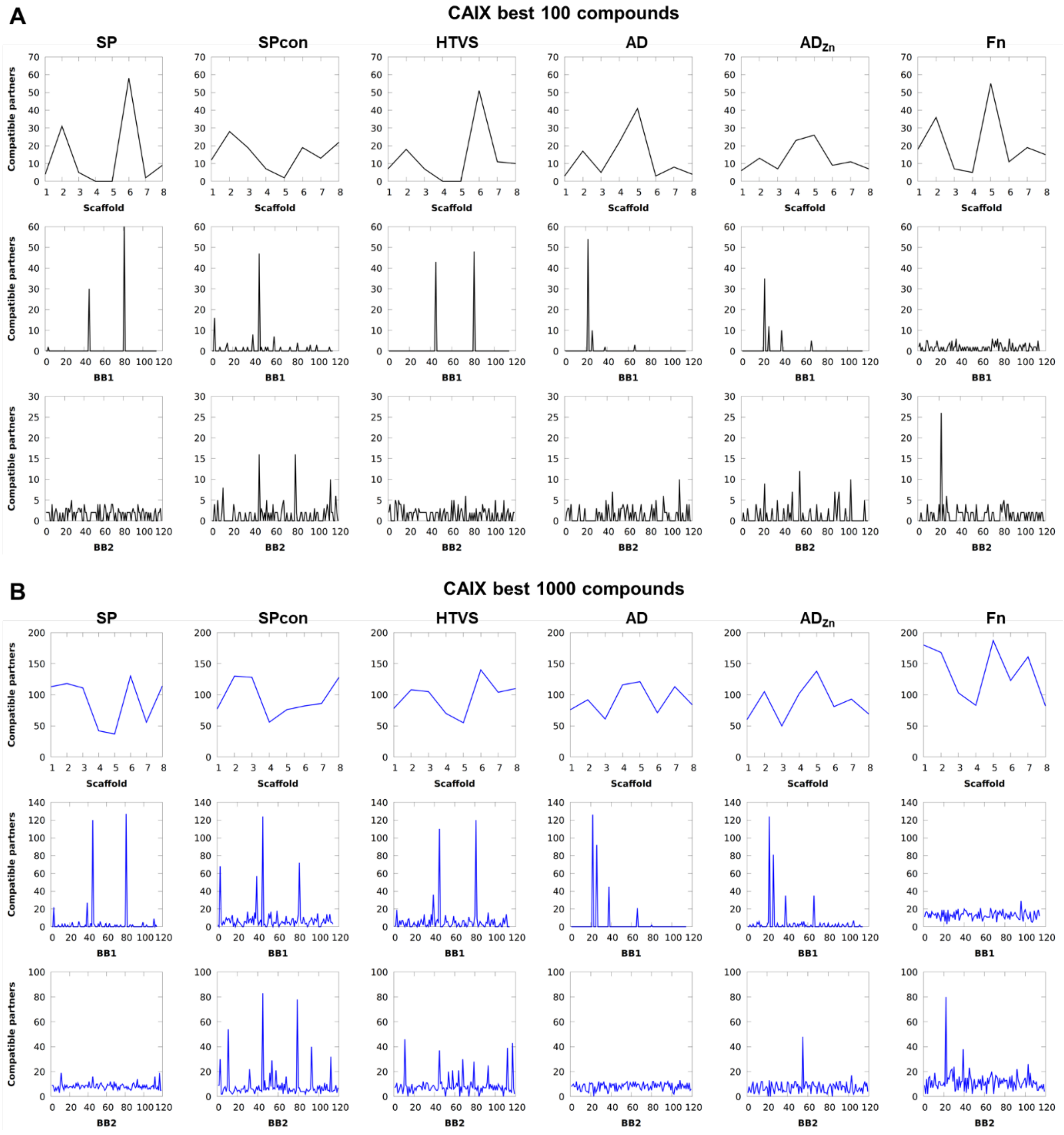

C CAIX best 13020 (number of exp. hits) compounds

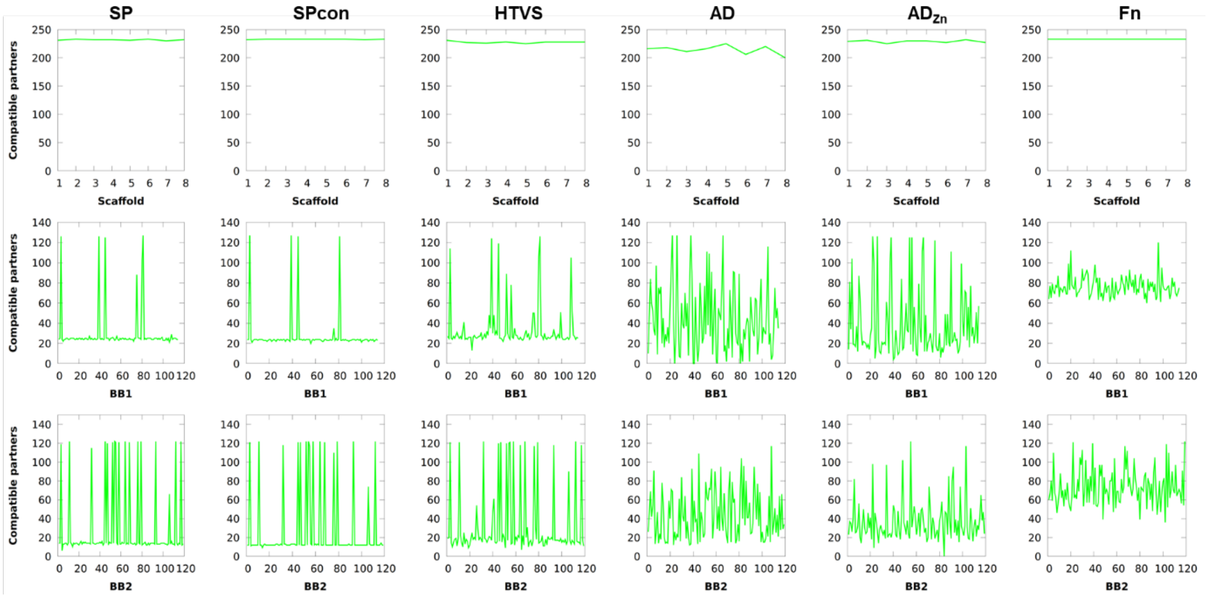

D HRP best 100 compounds

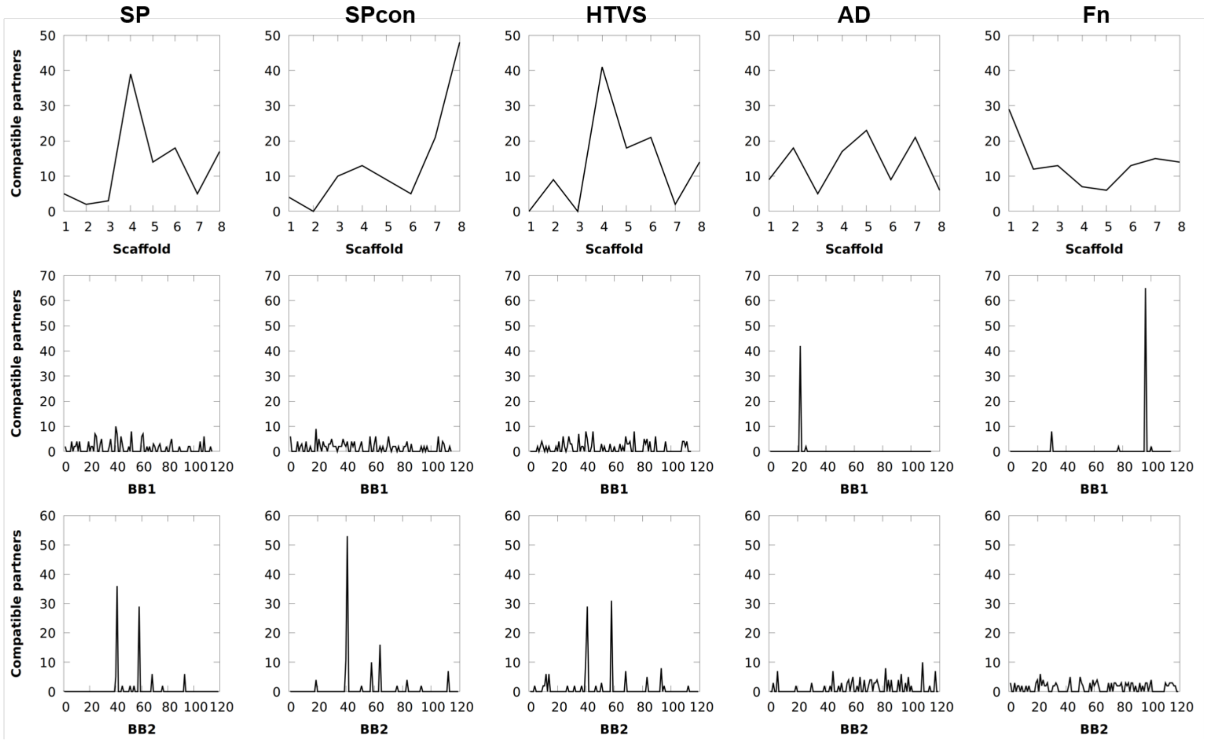

E

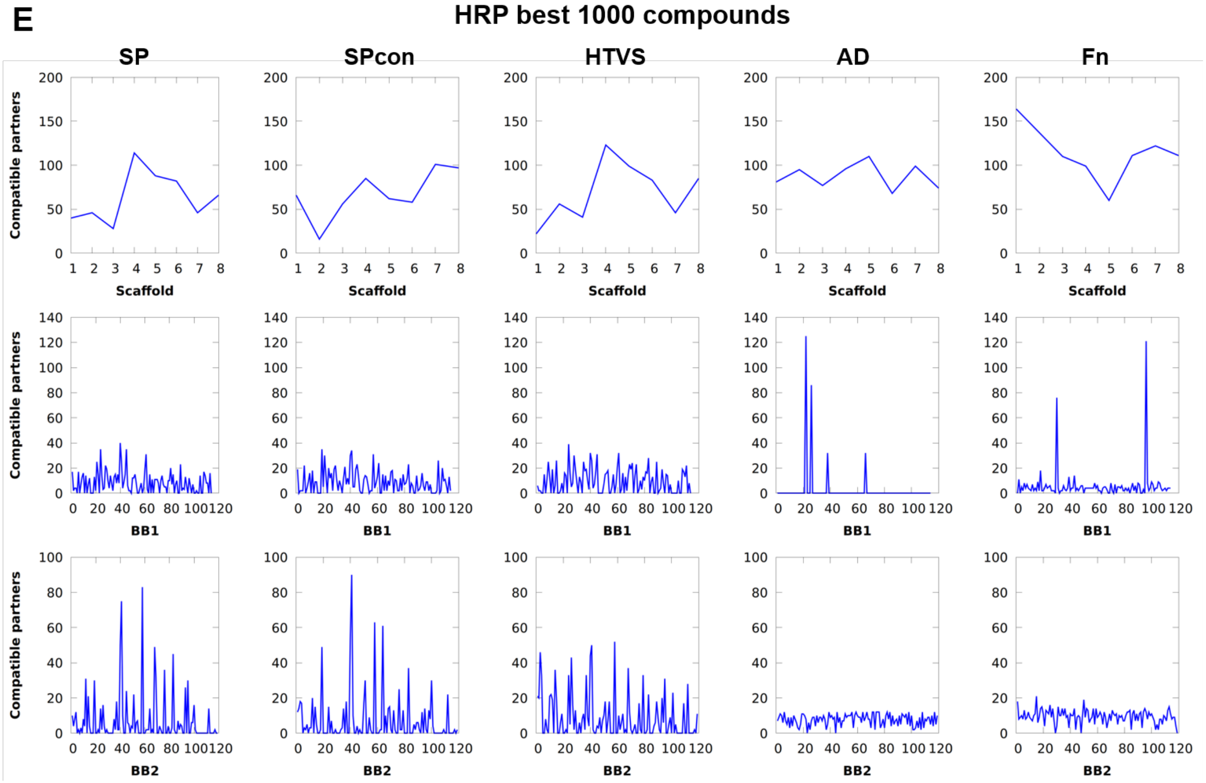
HRP best 1000 compounds
SP
SPcon
HTVS
AD
Fn
Compatible partners
Scaffold
BB1
BB2

F

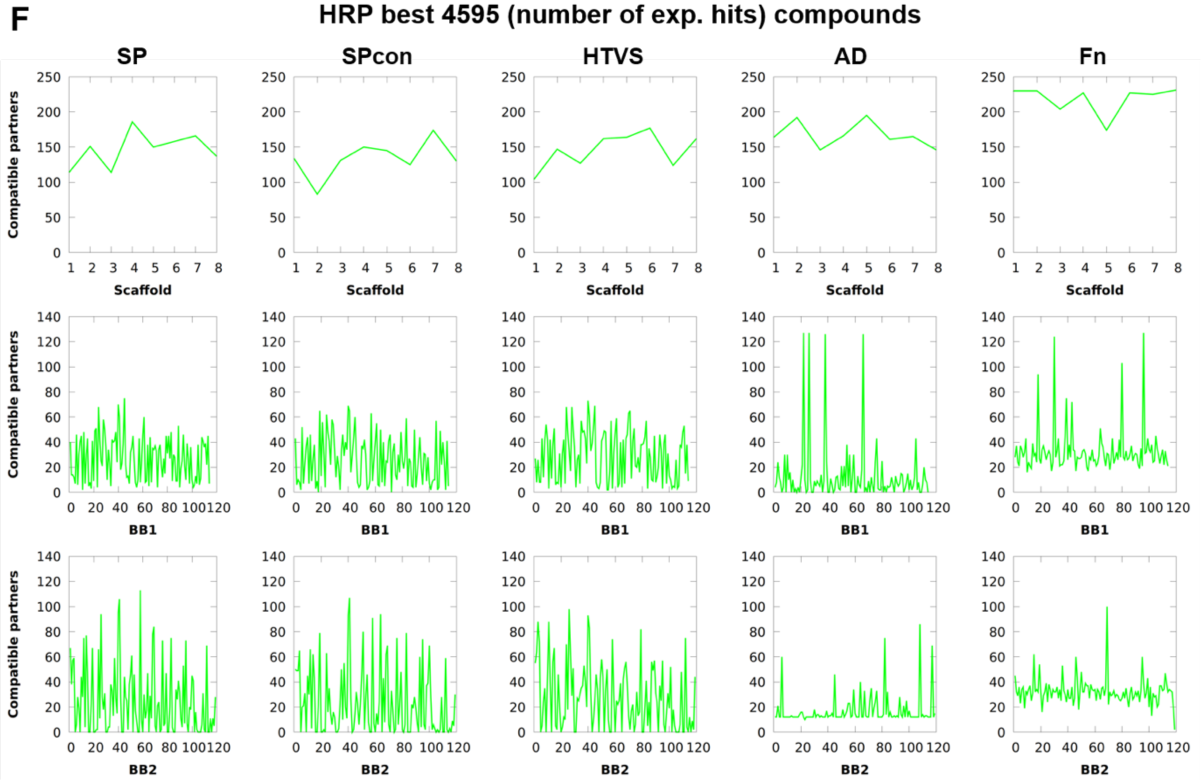
HRP best 4595 (number of exp. hits) compounds
SP
SPcon
HTVS
AD
Fn
Compatible partners
Scaffold
BB1
BB2

Figure S7. *Relation between normalized P_{bind} values and number of compatible partners for the best 1,000 compounds based on the SP, SPcon, HTVS and AutodockGPU docking scores and the Fn number against CAIX (A) and HRP (B). (black: scaffold | blue: BB1 | green: BB2)*

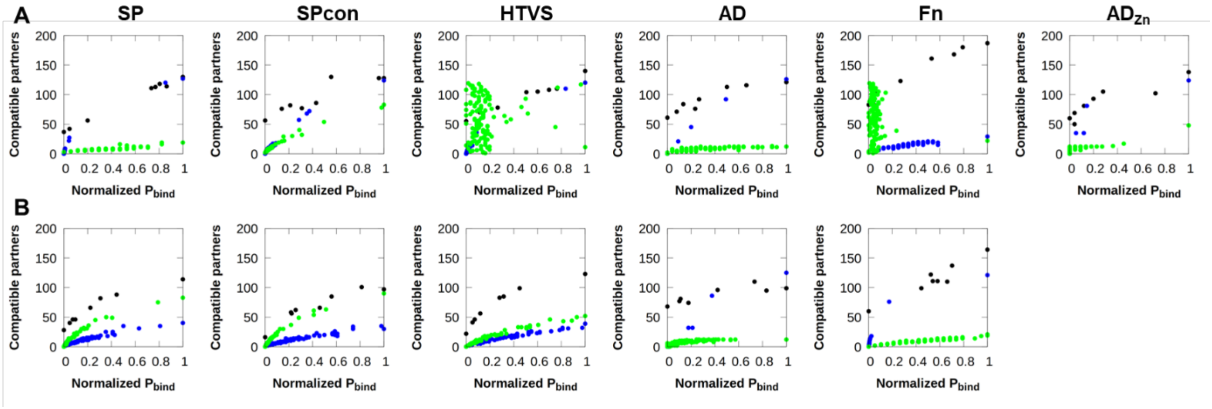

Figure S8. *Normalized P_{bind} values of the various building blocks at specific positions using the best 1,000 (black), 10,000 (blue) and 100,000 (green) DEL-A compounds based on the HTVS docking scores against MAPK (A) and AurA (B).*

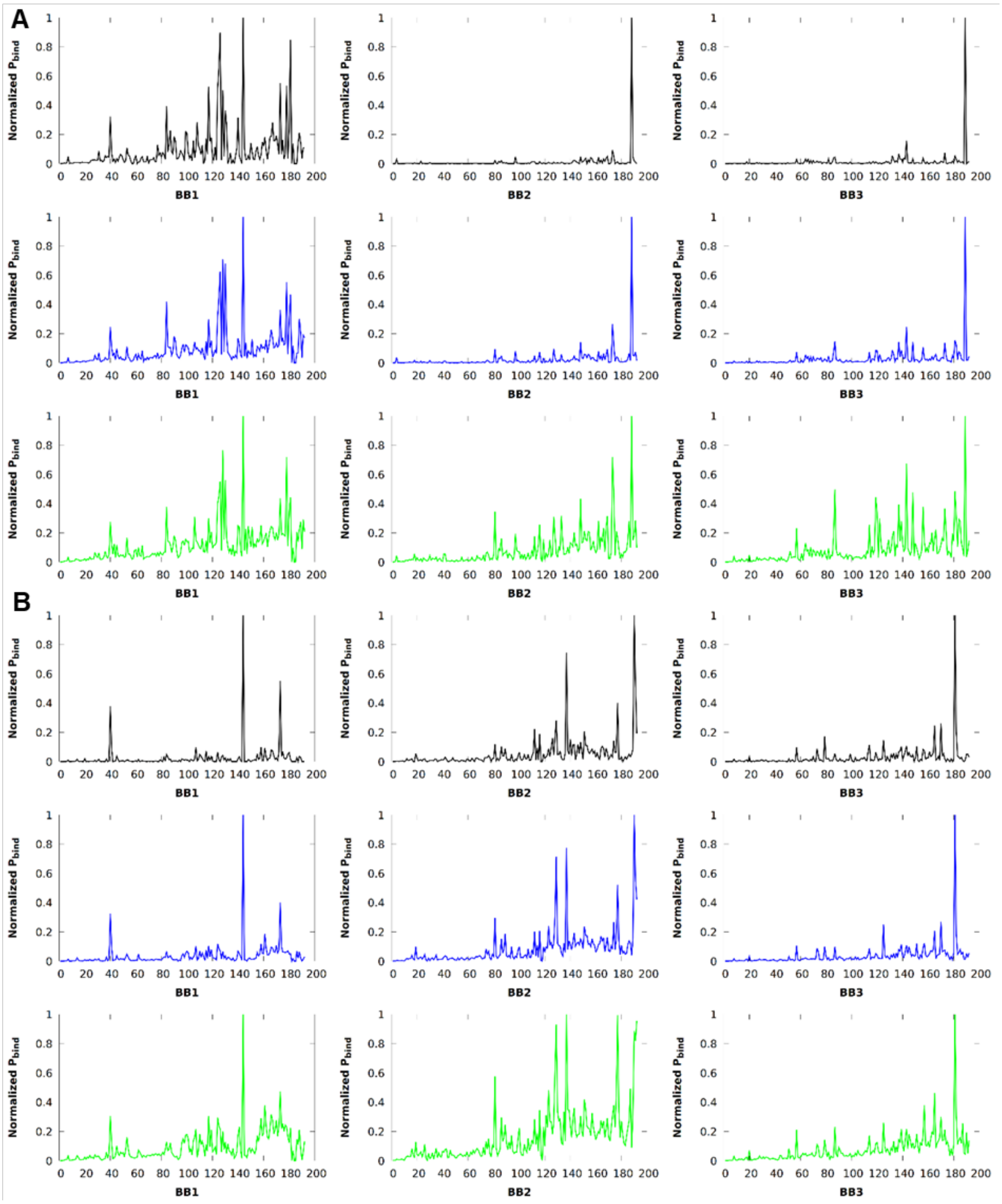

Figure S9. *Normalized P_{bind} values of the various building blocks at specific positions of the experimental DEL-A hit compounds against MAPK (A) and AurA (B).*

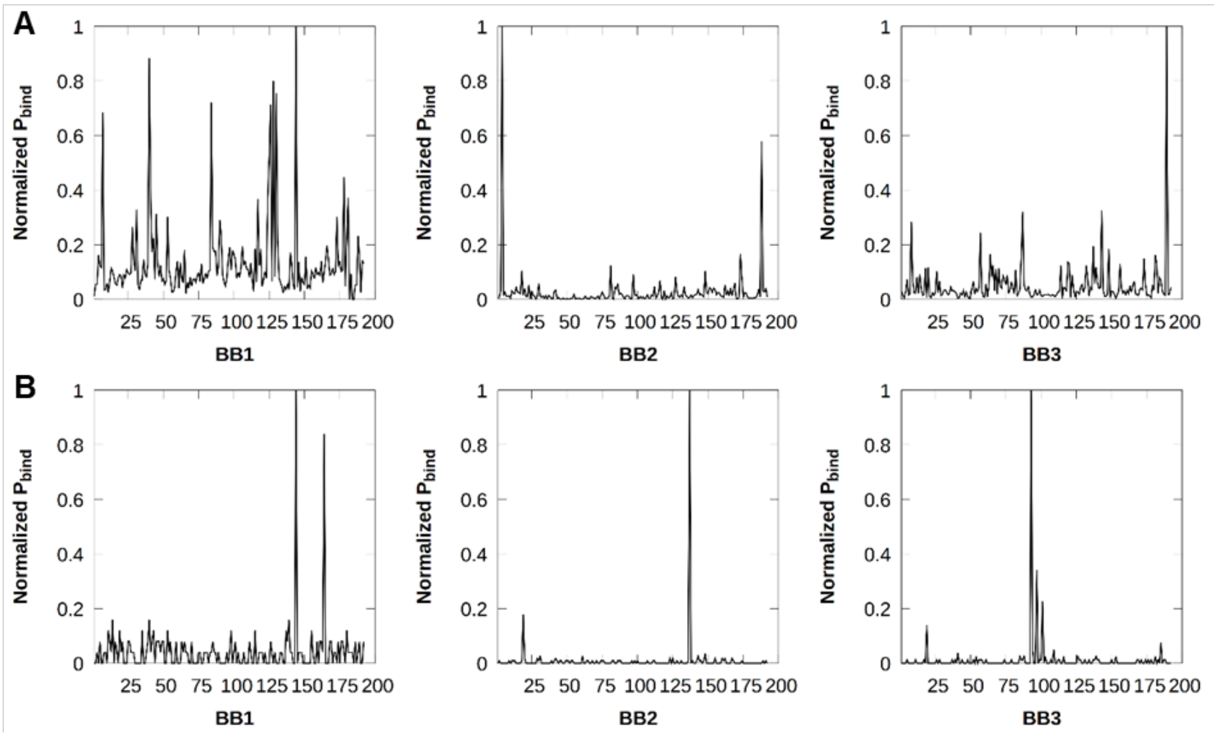

Figure S10. *Compatible partners of building blocks at specific positions using the best 1,000 (black), 10,000 (blue) and 100,000 (green) DEL-A compounds based on the HTVS docking scores against MAPK (A) and AurA (B).*

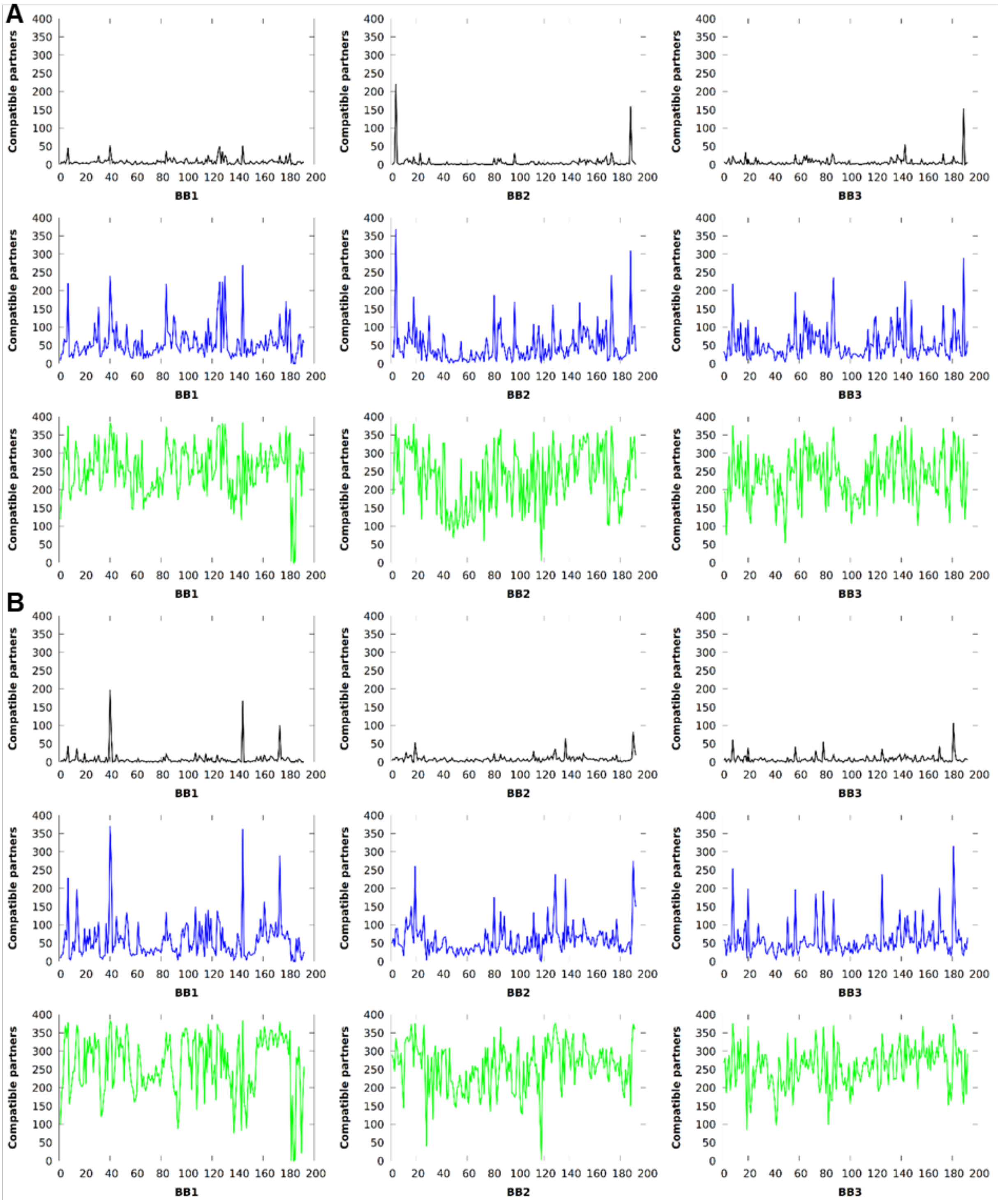

Figure S11. *Normalized P_{bind} values of the various building blocks at specific positions of the experimental DEL-A hit compounds against MAPK (A) and AurA (B).*

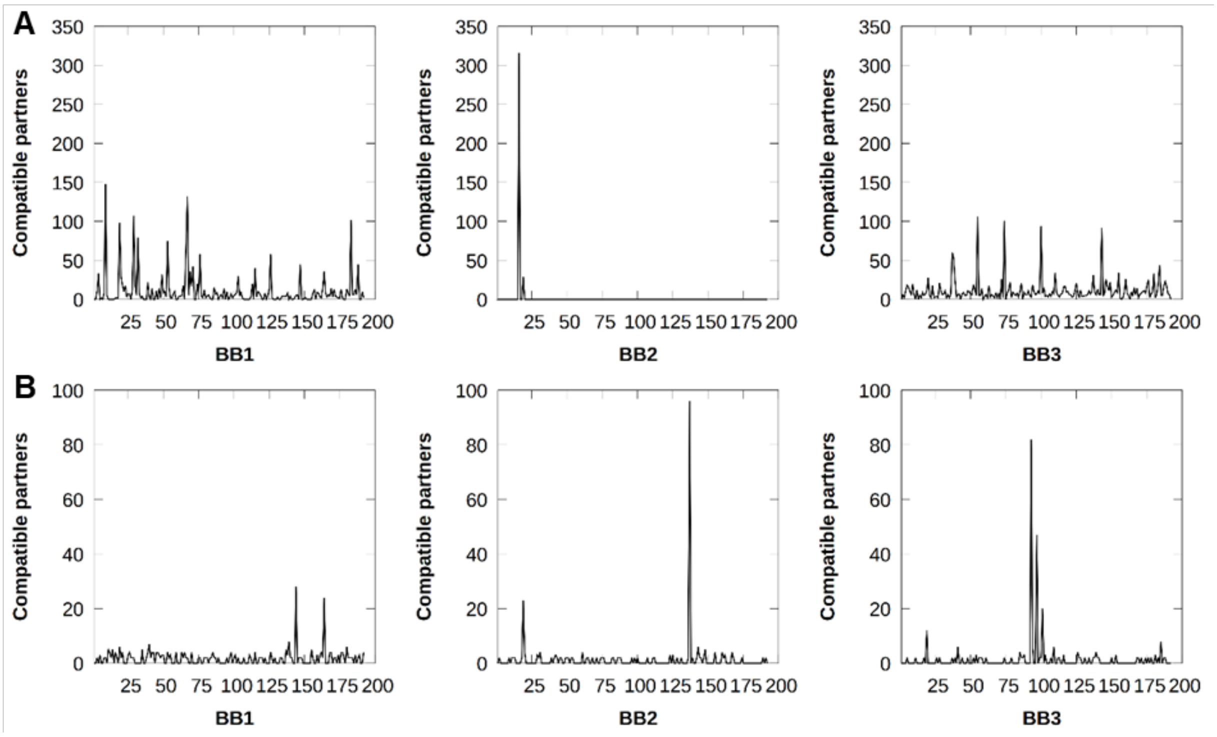

Figure S12. *Relation between normalized P_{bind} values and number of compatible partners for the best 1,000 compounds based on the HTVS docking scores and experimental hits against MAPK (A) and AurA (B) of the DEL-A set. (black: BB1 | blue: BB2 | green: BB3)*

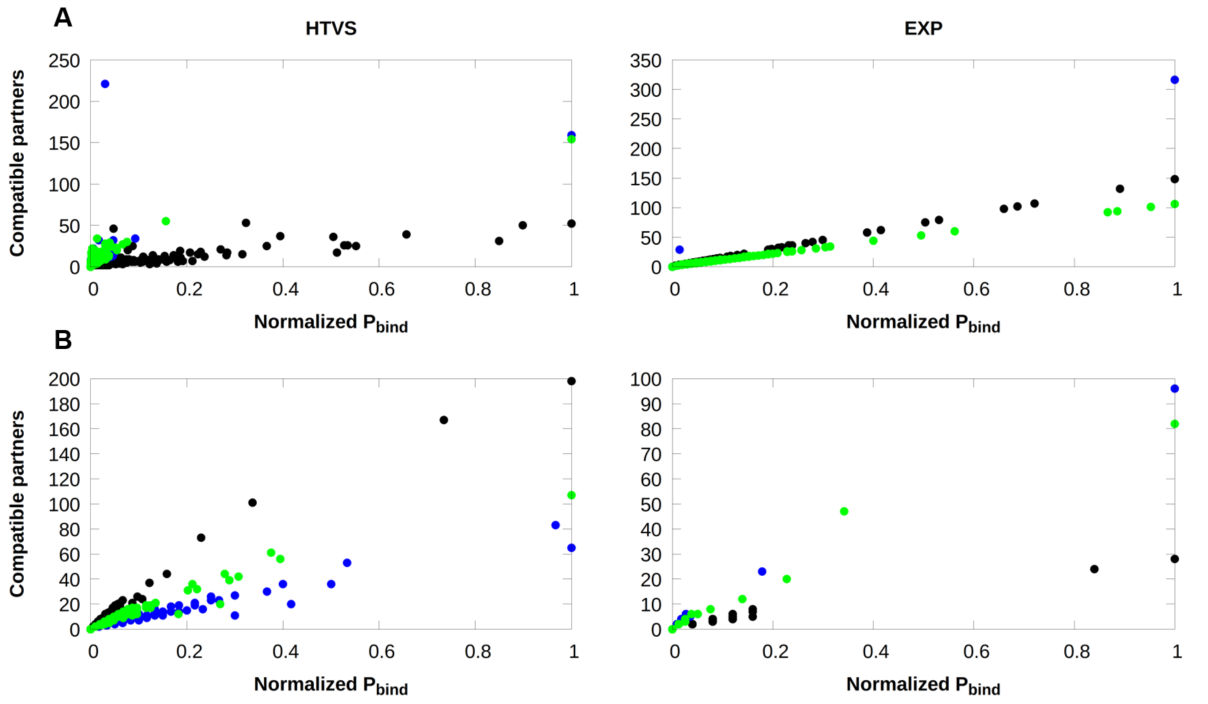

Figure S13. *Normalized P_{bind} values of building blocks at specific positions using the best 1,000 (black), 10,000 (blue) and 100,000 (green) DEL-B compounds based on the DeepDocking-Glide scores and the experimental hits (red) against MAPK.*

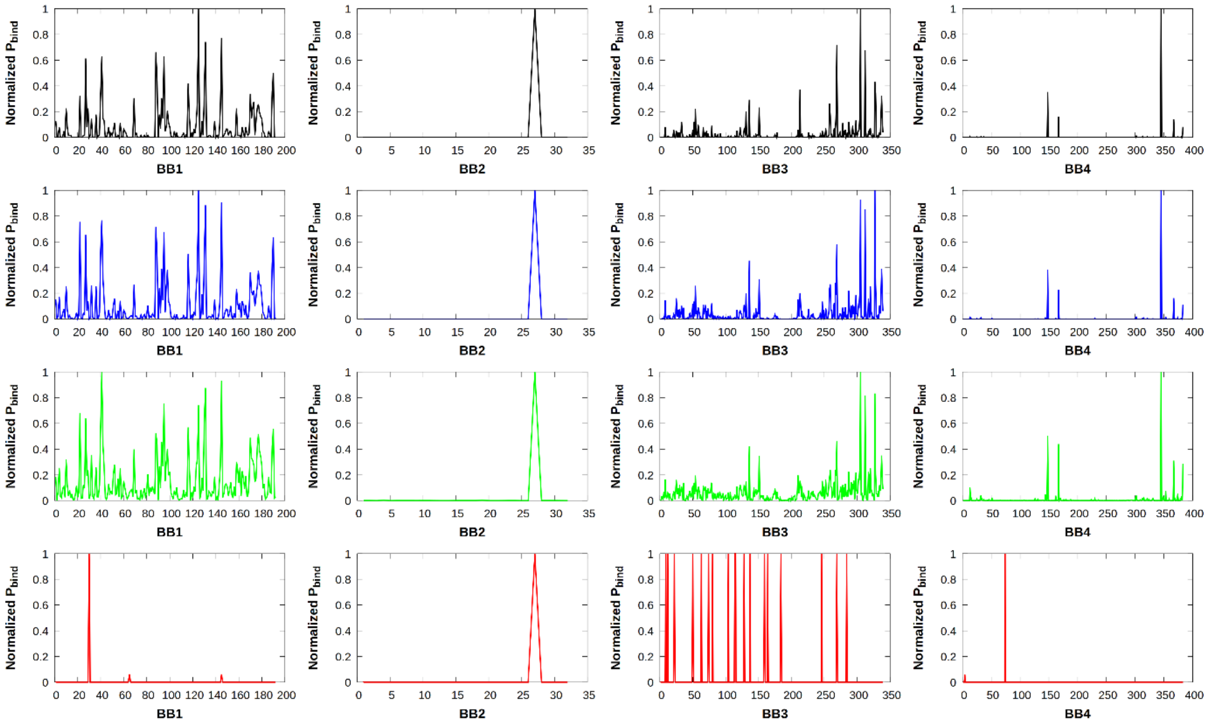

Figure S14. *Compatible partners of building blocks at specific positions using the best 1,000 (black), 10,000 (blue) and 100,000 (green) DEL-B compounds based on the DeepDocking-Glide scores and the experimental hits (red) against MAPK.*

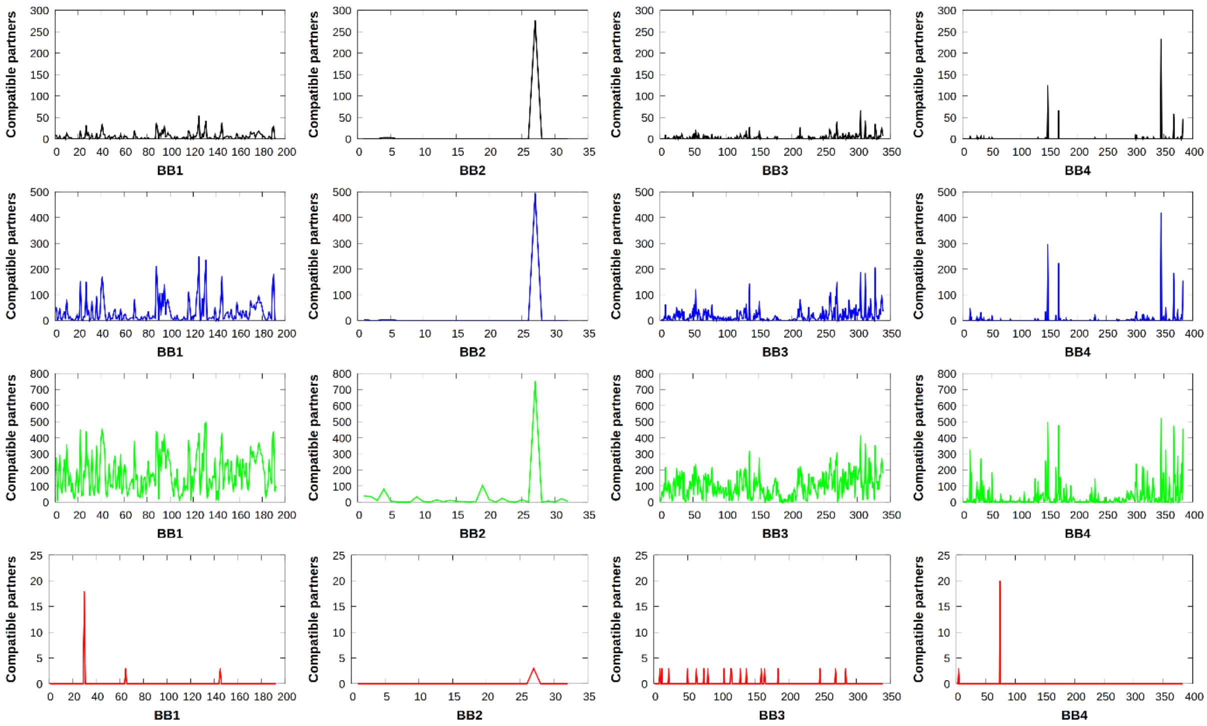

Figure S15. *Relation between normalized* P_{bind} *values and number of compatible partners for the best 1,000 compounds based on the DeepDocking-Glide scores and experimental hits against MAPK of the DEL-B set. (black: BB1 | blue: BB2 | green: BB3 | red: BB4)*

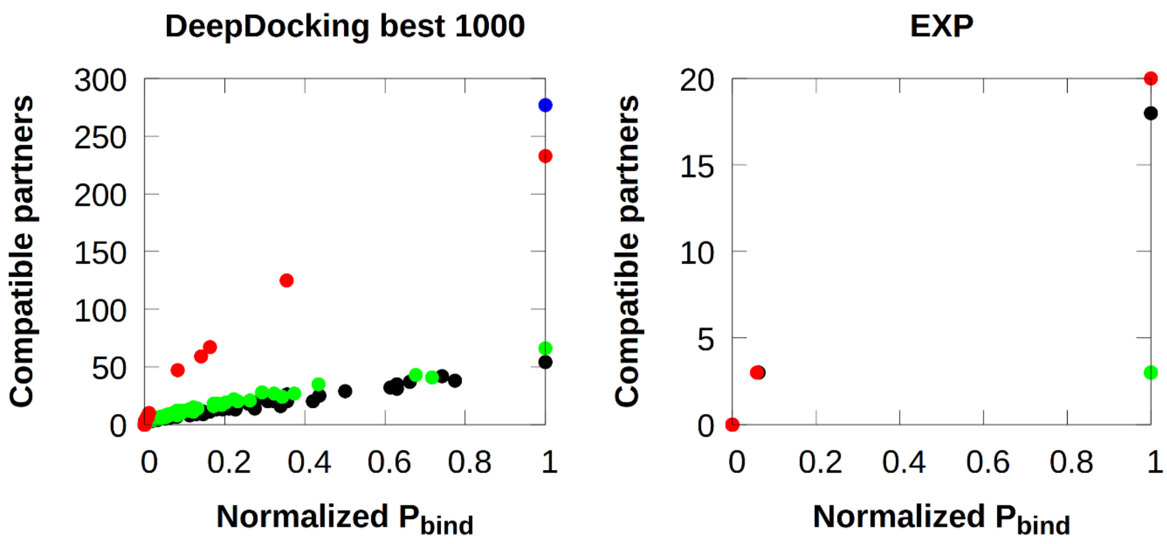

Figure S16. *Relation between the normalized* P_{bind} *values of the DEL-B experimental hits and the SP docking score of the individual building blocks against MAPK. (black: BB1 | blue: BB2 | green: BB3 | red: BB4)*

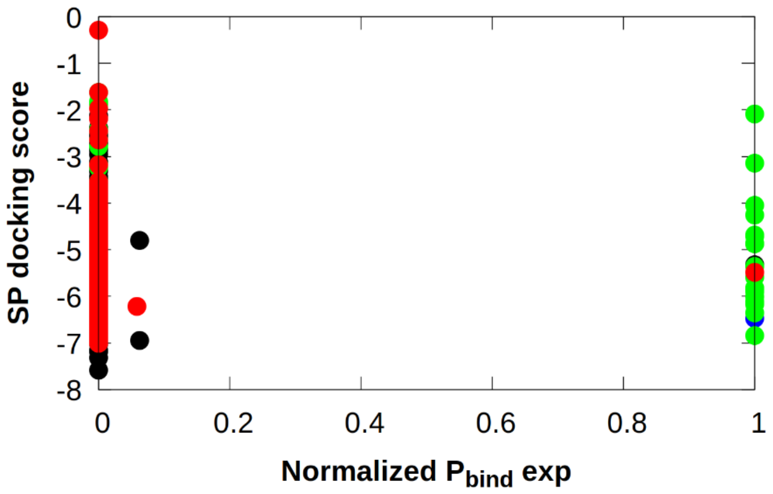

Figure S17. *ROC curves of the DEL-0 datasets after filtering out unfeasible poses.*

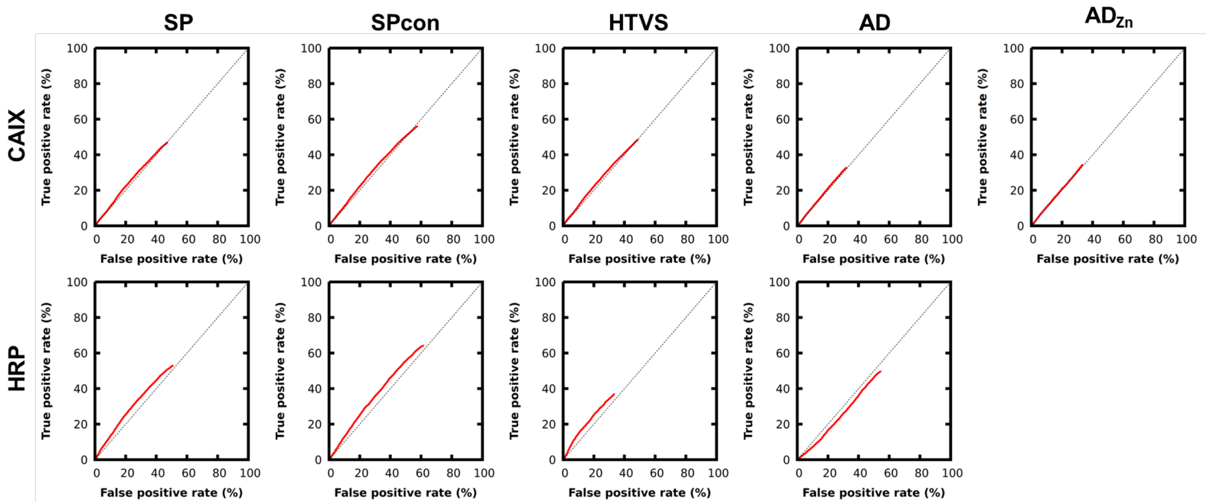

Supplementary notes

Supplementary Note 1. Benchmarking statistics of the DeepDocking workflows

To evaluate the goodness of the DD models, the benchmarking statistics (precision, recall, F_1-score and ROC-AUC) for each iteration were used and were plotted in **Figure S18**.

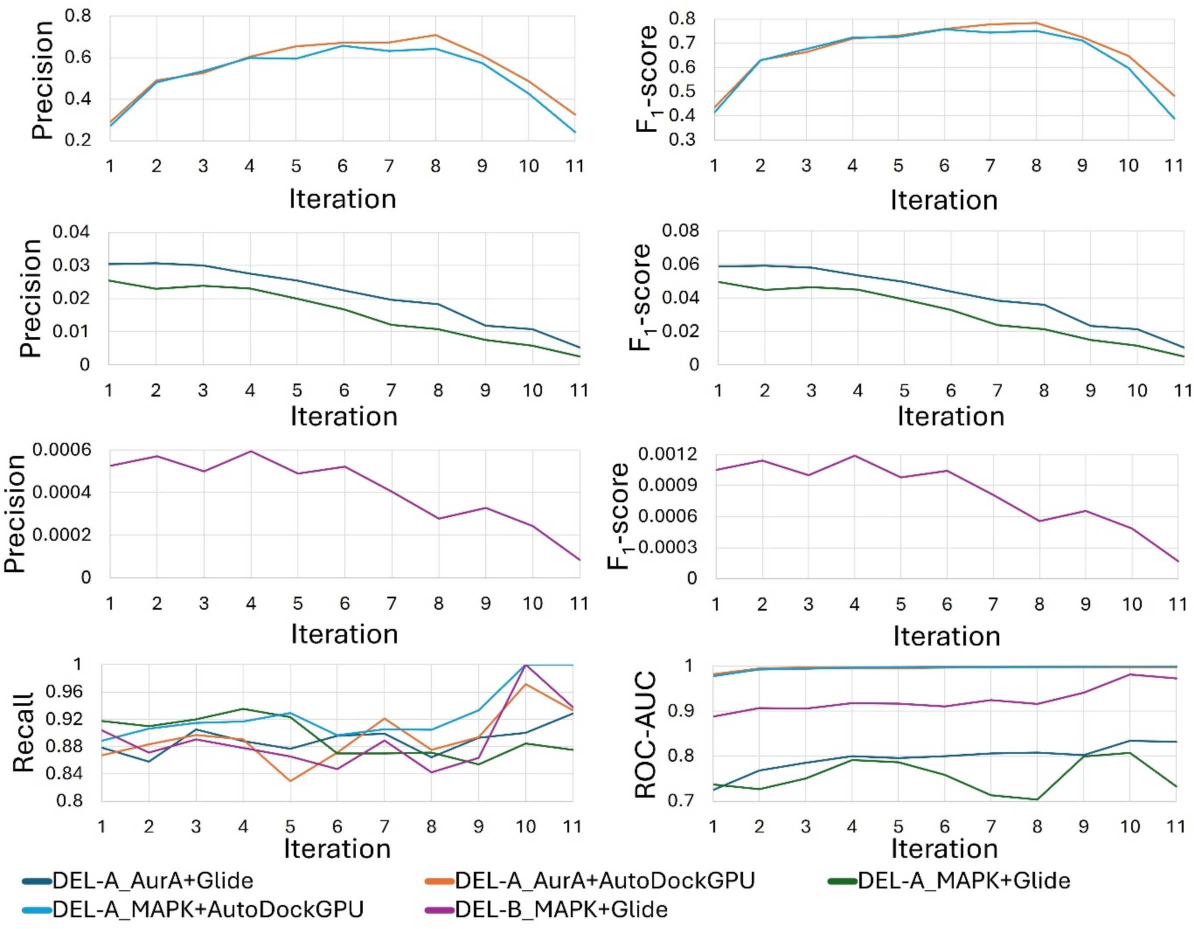

Figure S18. DeepDocking benchmarking statistics (precision, recall, F_1-score, ROC-AUC) for each run.

Recall values fluctuated close to the implicitly set 0.9 throughout the iterations, while precision and F_1-score values gradually decreased throughout all iterations for all DD runs done with Glide, and only decreased after the eighth iteration for runs done with AutoDockGPU. The decrease in precision suggests that, over iterations, the number of false positives is increasing. This means that while the model continues to identify a high number of true positives, it also increasingly misclassifies negatives as positives. In this case, it suggests that the model predicts increasing number of false positives throughout each iteration to reach a recall value of 0.9. The decreasing nature of the precision value with a relatively constant recall value is not unexpected

in the case of virtual screening, where there are usually way more true negatives in the dataset, than true positives, and the model might be tuned to ensure high recall by capturing all possible positives, thereby increasing false positives and reducing precision. Low precision and thus low F_1-score values in the case of Glide runs also explain the large number of prospected virtual hits and false positives.

In the case of the DD runs done with AutoDockGPU, the precision values stay above 0.2 across all iterations, while the F_1-score values stay between 0.4-0.8, both with a maximal value between the sixth and eighth iterations. Even though the model is more precise, it is only precise in predicting docking results from AutoDockGPU, which does not necessarily means being precise in identifying true positives (as DD performance depends on the suitability of the docking engine).

Another benchmark statistic worth considering is the ROC-AUC values, which should have an increasing tendency throughout the iterations, corresponding to an increasingly better model in discriminating positive and negative classes. This increasing tendency is observed in all runs, expect for the run done with Glide on MAPK using the DEL-A dataset, which has the lowest AUC value after iteration eleven, and does not show a clearly increasing tendency throughout the iterations.
